# Synthetic and analytical considerations for the preparation of amorphous metal–organic frameworks

**DOI:** 10.1039/d4sc01433b

**Published:** 2024-06-24

**Authors:** Emily V. Shaw, Ashleigh M. Chester, Georgina P. Robertson, Celia Castillo-Blas, Thomas D. Bennett

**Affiliations:** a Department of Materials Science & Metallurgy, University of Cambridge 27 Charles Babbage Road Cambridge UK tdb35@cam.ac.uk

## Abstract

Metal–organic frameworks (MOFs) are hybrid porous materials presenting several tuneable properties, allowing them to be utilised for a wide range of applications. To date, focus has been on the preparation of novel crystalline MOFs for specific applications. Recently, interest in amorphous MOFs (aMOFs), defined by their lack of correlated long-range order, is growing. This is due to their potential favourable properties compared to their crystalline equivalents, including increased defect concentration, improved processability and gas separation ability. Direct synthesis of these disordered materials presents an alternative method of preparation to post-synthetic amorphisation of a crystalline framework, potentially allowing for the preparation of aMOFs with varying compositions and structures, and very different properties to crystalline MOFs. This perspective summarises current literature on directly synthesised aMOFs, and proposes methods that could be utilised to modify existing syntheses for crystalline MOFs to form their amorphous counterparts. It outlines parameters that could discourage the ordering of crystalline MOFs, before examining the potential properties that could emerge. Methodologies of structural characterisation are discussed, in addition to the necessary analyses required to define a topologically amorphous structure.

## Abstract 2

Amorphous MOFs (aMOFs) are often prepared through introducing disorder to crystalline frameworks. This perspective aims to introduce directly synthesised aMOFs, expanding on synthetic methods, characterisation techniques and potential properties.

## Graphical abstract

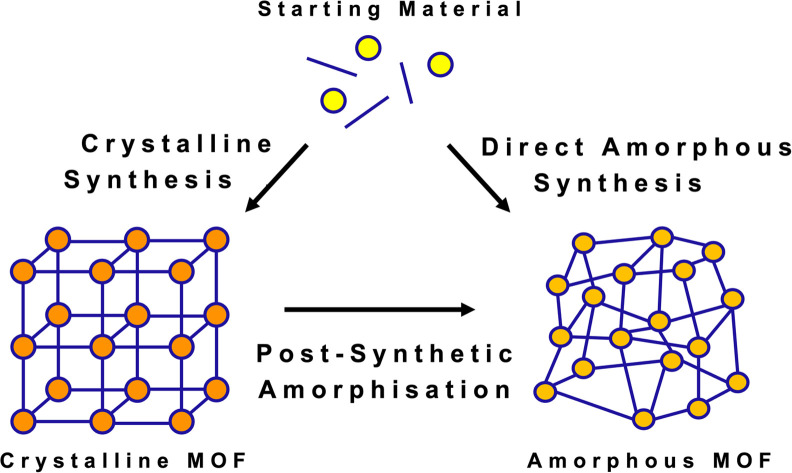

## 1. Introduction

Since their discovery in the 1990s,^1,2^ the last 30 years have seen the emergence of metal–organic frameworks (MOFs) as a new classification of porous materials, and the subsequent development of the concept of reticular chemistry.^3–7^ Formed through the self-assembly of metal nodes, known as secondary building units (SBUs), and organic ligands or linkers, these three-dimensional networks have a wide range of porosities and topologies.^8–10^ Owing to their tuneable functionality, high surface area, permanent porosity and high chemical stability,^11–13^ these materials have emerged as promising candidates for several applications such as catalysis,^14,15^ gas sorption,^16–19^ drug delivery^20–23^ and water harvesting.^24–26^ Since their discovery, interest in these materials has grown exponentially, with >100 000 structures currently reported in the literature.^27–29^ Despite this, MOFs have multiple intrinsic challenges, including low mechanical stability,^30^ non-sustainable synthesis conditions and problems surrounding the scalability of their synthetic procedures.

**Fig. 1:**
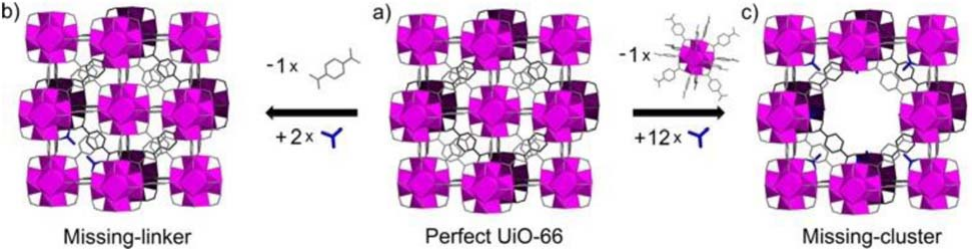
Idealised representations of defective UiO crystalline frameworks. (a) Crystalline, non-defective framework, (b) replacement of one linker with monocarboxylic groups, (c) replacement of one metal cluster with monocarboxylic groups. Zr_6_O_8_ polyhedr node – pink. Reproduced from Taddei *et al.* with permissions from Elsevier 2017.^32^

The inclusion of defects within ordered MOF structures has been observed to alter the chemical and physical properties of these crystalline systems, presenting an exciting avenue for reticular design. Defect engineering, defined as the controlled introduction of defects within a crystalline structure, presents an additional way to modify a materials functionality. Defects in MOFs can be classified into: missing linker (linker position vacant within the structure), missing node (metal-cluster position vacant within the structure) or modified node/linker (metal-cluster or ligand exchanged to other moiety) as is depicted in Fig. 1. These are defined as point defects, those which have zero dimensionality within the lattice. It is these defects which have been identified as the active sites for several applications, such as catalysis and gas sorption.^31^

The literature delineates two primary methods of defect engineering: *de novo* (from scratch) synthesis, involving the deliberate introduction of defects during synthesis, and post-synthetic modification, applied to pre-synthesised MOFs.^33^ Post-synthetic introduction of defects primarily utilises partial thermal decomposition of the linker, known as decarboxylation, whilst maintaining the parent MOF structure.^34^ These defects were found to increase the number of accessible metal sites, resulting in improved catalytic activity and porosity.^35–37^ Given that partial linker decarboxylation was observed with classical carboxylate linkers, common within MOF structures, this methodology could be applied to a wide range of materials.^35,36^ Alternatively, post-synthetic ligand exchange, utilising monocoordinated linkers, has been observed to facilitate the introduction of point defects to the crystalline material.^38^

The introduction of defect sites through the *de novo* synthesis of defective MOFs has primarily been observed through the introduction of missing-linker defects through the use of competitive linkers, those which preferentially bind over that of the MOF-linker. Several monocarboxylic linkers have been utilised, including traditional modulators (benzoic acid, formic acid, among others) used to prepare more crystalline materials and competitive linkers with varying degrees of coordination (*i.e.* synthesis of a MOF utilising a mixture of tritopic and tetratopic linkers).^39–43^ The concentration of defects within the structure has been found to be tuneable through both acidity and concentration of this competitive linker.^44–46^ Additionally, the solvent medium has also been shown to influence the defect concentration. For example, water as a solvent influenced not only the topology of the UiO-based MOF, but also acted, along with hydroxide, as a capping linker to introduce MOF defects.^47,48^ Varying the concentration of water within the synthetic medium can therefore be utilised to tune the defect concentration.^47^ Furthermore, DMF, a prevalent solvent in MOF synthesis, induces controllable formate defects within MOF-74, [M_2_(dot)] (dot = 2,5-dioxidoterephthalate), with the defect concentration dependant on the metal : linker ratio.^49^

Build-up of defects to a critical concentration (the threshold of defect number which results in a drastic decrease in crystallinity) has been observed to result in the collapse of the structure. This results in a highly disordered material, known as an amorphous material, which likely still possesses several interesting properties.^46,50^

**Fig. 2:**
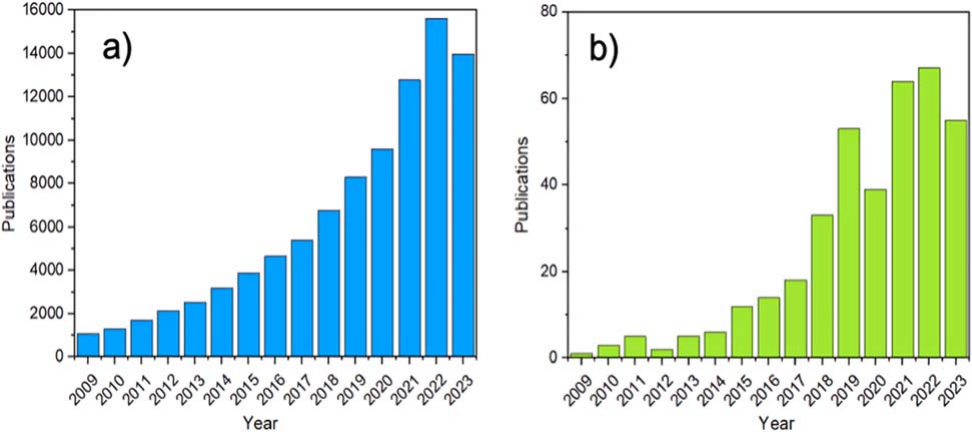
The number of MOF publications from 2009 to 2023. (a) The search terms ‘MOF’ or ‘metal–organic framework’, (b) the terms ‘amorphous’ or ‘amorphisation’ were set as a requirement to feature in the abstract, were used to search Web of Science.

In recent years, increased attention has been directed at amorphous MOFs (aMOFs), shown through the growing number of papers published yearly (Fig. 2). aMOFs are defined by their lack of long-range order (LRO, >∼7 Å), whilst still retaining extended connectivity and short-range order (SRO), often comparable to equivalent crystalline MOF (cMOF) systems.^51,52^ We do not suggest this latter abbreviation is more widely used, though the importance of the distinction between amorphous and crystalline phases in this review means it will be used here for clarity.^6,53–55^

These materials combine the advantageous properties of cMOFs with those of amorphous materials, including increased processability, with respect to microcrystalline powders, and higher intrinsic defect concentrations.^6,56–58^ These intrinsically defective aMOF systems present improved electronic, catalytic and mechanical functionalities due to the presence of high energy sites.^59^

This has resulted in applications in gas separation,^60,61^ catalysis^62,63^ and drug delivery.^64^ Surprisingly, these materials remain scarce, with only ∼200 structures reported.^61,65^ This is commonly attributed to their challenging structural characterisation limiting the ability to rationally design these materials for specific applications.

### 1.1. Classification of ‘amorphous’ MOF structures

**Fig. 3:**
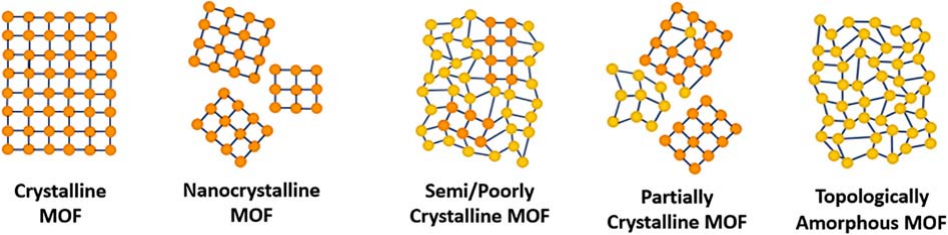
A 2D representation of the difference in long-range order between crystalline, amorphous and poorly/semi-crystalline MOFs. Orange/yellow spheres represent metal clusters/SBUs and blue lines represent organic linkers.

Within the literature, there are several ‘degrees’ of crystallinity reported, with limited differentiation made between these phases. This perspective aims to highlight distinctions between these structures and provide thoughts on the general (mis)classification of crystalline, defective, and amorphous structures. Idealised MOF structures are displayed in Fig. 3.

Whilst literature does frequently attempt to differentiate crystallite size, the degree and nature of disorder in a sample is less commonly defined. An idealised single crystal MOF would not contain defects within the ordered structure, however, experimentally, a degree of defectiveness in the structure is inevitable. Decreasing the crystallite size of a given material, without altering defect concentration, can lead to its classification as nanocrystalline, often when crystallites are ∼<20 nm in diameter. Conventional spectroscopic techniques, *e.g.* X-ray diffraction, are often unable to distinguish between the effects of a change in particle size from a change in the level of sample disorder. This often leads to challenges in material phase identification, preventing distinction of a nanocrystalline phase from an amorphous phase, (discussed later).

A semi- or poorly crystalline MOF material will henceforth be defined as a material possessing interconnected regions of both localised crystallinity or order extending into the longer range, and disorder within its interconnected structure. While it may be viewed as a highly defective crystalline material, the presence of disorder within the structure is often visible from common characterisation techniques but not quantifiable with respect to the crystalline components. The introduction of defects, likely missing node/linker or displacement defects, to a crystalline structure can result in a transition to a semi/poorly crystalline material, but with some retained connectivity, before potentially subsequently collapsing to an aMOF.

We propose that the term ‘partially crystalline MOF’ is frequently used in the literature to indicate a sample containing disconnected particles of both amorphous and crystalline structures, representing the sample as a whole. Here, we use it to describe literature samples where no definitive conclusion can be drawn from the available evidence. In contrast, a topologically amorphous MOF retains no correlated order at extended length scales, but retains extended connectivity associated with the crystalline equivalent. Henceforth, when an amorphous material is referenced within this perspective, we refer to this topologically amorphous model. We theorise that there are two types of potential aMOFs which could form. One retains the SRO of the equivalent crystalline material, whereas the other possesses a modified local structure. In both cases, the amorphous nature of the MOF is defined by the lack of extended order.

Several reviews exist in the literature, covering a wide scope of both crystalline and amorphous MOFs.^66–70^ Reviews into aMOFs to date have focused primarily on post-synthetic amorphisation of a crystalline parent material. In contrast, this review aims to focus on synthetic control to directly synthesise an aMOF (*i.e.*, without initially producing a crystalline material), termed a_s_MOF, and speculates on the potential application of these materials. Additionally, focus is directed at the utilisation of a range of analytical techniques to differentiate between the structures and level of disorder displayed in Fig. 3.

## 2. Synthesis of aMOFs

**Fig. 4:**
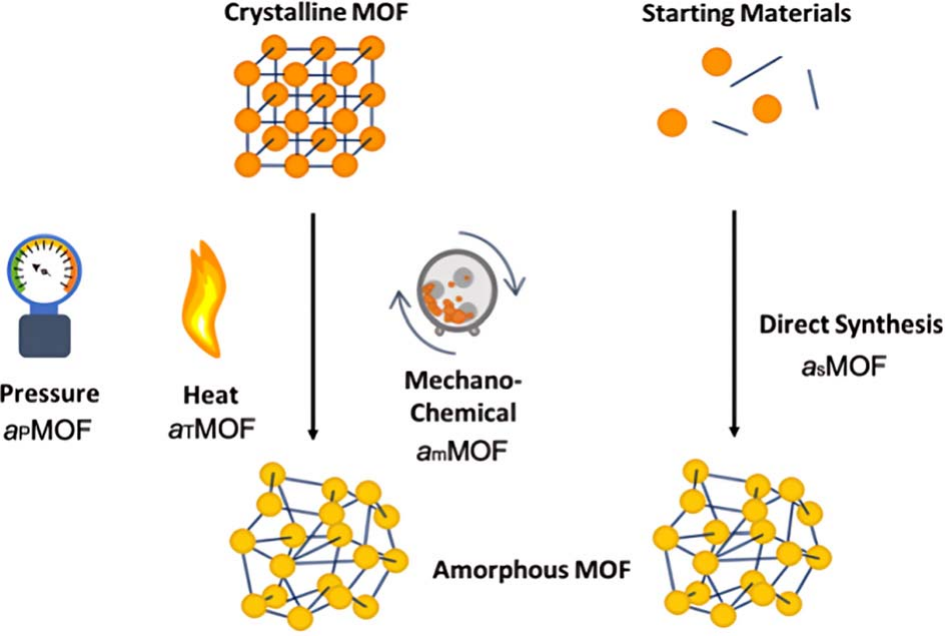
Methodologies for the formation of aMOFs. Orange/yellow spheres represent metal clusters/SBUs, and blue lines represent organic linkers. Notations will be used throughout, with a_P_ defining pressure induced amorphisation, a_T_ thermal amorphisation, a_m_ mechanochemical amorphisation and a_S_ directly synthesised amorphous materials.

aMOFs can be fabricated through different synthetic approaches (Fig. 4), with most focusing on the post-synthetic collapse of the corresponding crystalline parent material. This may be achieved by the application of heat,^53,67,71–74^ pressure,^75–79^ or shear stress,^80–84^ which causes either atom displacement or successive introduction of other defects through bond breakage (and sometimes reconstruction) to a critical concentration, resulting in a total or partial collapse of the LRO.

An emerging and challenging alternative to structural collapse for the generation of a topologically amorphous MOF structure is through direct synthesis utilising similar precursor materials to those employed during the synthesis of cMOFs. Direct synthesis has been observed to preserve both the intrinsic and extrinsic pore structure of the MOF, commonly lost during alternative amorphisation methods.^6,53,54^

### 2.1. Post-synthetic collapse

Literature reports several post-synthetic methods of amorphisation. These include, but are not limited to, ball-milling utilising shear stress (mechanical amorphisation, a_m_), pressure (pressure-induced amorphisation, a_P_), heat (thermal amorphisation, a_T_) and chemical methods. As shown in Fig. 4, post-synthetic amorphisation requires the preparation of a crystalline starting material, often the most time-intensive and challenging stage of the aMOF preparation. Whilst there have been some examples of aMOFs being directly prepared by ball-milling starting reagents, these reactions proceed through a crystalline intermediary.^85^ This, in addition to the often energy and time intensive processes involved, limit the utilisation of these amorphisation methods for industrial implementation.

Investigation into the mechanism of structural collapse of different materials identified progression through propagation of defect introduction into the structural network, often through breakage of weak node-linker bonds within the MOF structure. However, the exact mechanism of collapse is highly dependent on the nature of the MOF and consequently limits the predictability of either the structure or properties of the resultant aMOF.^86^ An additional challenge is that resultant aMOF structures are often classified as non-porous, as a result of a collapsed microporous structure.^87^

For example, investigation of a_p_ZIF-8, [Zn(mIm)_2_] (mIm = 2-methylimidazolate), noted a large decrease in Brunauer–Emmett–Teller (BET) surface area compared to the crystalline parent material, suggestive of a partial collapse of the microporous structure. This retention could potentially be attributed to partial recrystallisation of the sample after pressure was removed.^88^ This highlights the challenge of preparing a truly amorphous material with retained porosity.

Whilst solvent templating has been suggested as a route to preserving the porosity of the MOF during amorphisation, this can result in an increase in the elastic moduli of the material through solvent occupancy in the pore structure, likely resulting in resistance to amorphisation even under more extreme conditions.^89^ The challenge of this was noted through pressure-induced amorphisation of solvated ZIF-4, [Zn(Im)_2_] (Im = imidazolate), where generation of an amorphous structure occurred at higher pressures compared to that of the evacuated sample.^78^ Elsewhere, ball-milling of solvent-stabilised MIL-100, [Fe_3_O(H_2_O)_2_OH(btc)_2_] (btc = 1,3,5-benzenetricarboxylate), for short periods of time was observed to increase the BET surface area relative to the evacuated sample.^90^ Whilst the ball-milling time was insufficient to produce an amorphous sample, extended milling of a solvent-stabilised crystalline material may therefore preserve some porosity in the a_m_MOF. On the other hand, mechanochemical amorphisation of evacuated MIL-100 revealed a complete loss of accessible BET surface area, consistent with near-complete pore collapse.^90^

In literature, the term ‘thermal amorphisation’ often describes numerous amorphisation pathways, all requiring the application of heat to facilitate loss of structural order. One such mechanism involves the thermal activation of a cMOF, through heating under dynamic vacuum to remove solvent molecules from the pores. An illustrative example was reported with fast removal of MeOH from the pores of Mn-MIL-100, resulting in a collapse of the structure.^91^ Alternatively, there have been several examples of MOFs melting upon exposure to high temperatures to form a liquid phase, with fast quenching of this phase resulting in the formation of a melt-quenched glass (MQG).^92^ Literature often uses the term ‘glassy’ interchangeably with ‘amorphous’, however classification of a glass is only true if the amorphous material has a glass transition temperature (*T*_g_). At this temperature, an amorphous material transitions from a hard, brittle state to a rubbery, flexible state as it is heated. Whilst alternative amorphisation methods commonly produce a thermodynamically stable structure, melt-quenching can, in some cases, trap the structure in a different, kinetically-favoured form.

Thermal amorphisation, assumed to involve the breakage and reformation of M–L bonds (M = metal cation, L = organic ligand), is limited to certain MOFs because of the high enthalpy of crystallisation and structural reordering, relative to their low decomposition temperatures, *T*_d_.^34^ The same challenge is observed with forming MQG, as the melting temperature, *T*_m_, of MOFs is often greater than their temperature of decomposition. Several a_g_MOFs are reported in the literature, with a large majority based on zeolitic imidazolate frameworks (ZIFs).^57,92–98^ Control of the melting temperature of these MOFs, with the aim of reducing the *T*_m_ below that of the *T*_d_, has been achieved through the incorporation of bulky, flexible, weakly coordinating or low symmetry ligands to non-melting MOFs, or through the introduction of an ionic liquid.^92,93,99,100^ The presence of the ionic liquid stabilises the de-coordinated linker, facilitating the breakage of M–L bonds and lowering the enthalpy of melting.^99^ Amorphisation, through alternative methodologies, has been shown to lower the *T*_m_ of a MOF, allowing for subsequent quenching into an a_g_MOF material.^101,102^ In addition, these glassy materials have also been prepared without requiring melting, utilising ball-milling^85,103^ or desolvation^104^ to form amorphous materials displaying a *T*_g_. Interestingly, mechanochemical amorphisation has been observed to form glassy materials with both melting and non-melting MOFs, showing the wide potential of this methodology.^103^ Mechanochemical amorphisation of a range of ZIF-62, [Zn(Im)_2−*x*_(bIm)_*x*_] (bIm = benzimidazole), materials revealed changing the bIm ratio affected if a *T*_g_ was observed, with *x* = 0.35 the first time the mechanochemically induced *T*_g_ was noted.^85^ Understanding why these materials present a *T*_g_ could allow for design of a_s_MOF materials displaying the same glassy properties. Additionally, the synthetic considerations outlined by Wei *et al.* for the formation of glassy MOFs upon desolvation could be applied to further aMOF materials.^104^

Glassy MOFs represent an important emerging field within aMOFs, having been observed to not only possess lack of grain boundaries, but also tuneable porosity which could be exploited for gas separation.^105^ In the literature, there are several reviews on the topic published to date.^53,106–108^ Because of this, these materials will not be explored further.

Structural collapse has also been triggered through chemical processes. Similar to defect engineering, the introduction of competitive ligands or metals to a crystalline MOF material by chemical methods can increase defect concentration enough to induce amorphisation. The concept of competitive linker binding has been demonstrated to induce crystalline-amorphous transitions for MIL-88B,^109^ [Fe_3_O(NH_2_-bdc)_3_], MIL-68-NH_2_,^62^ [In(OH)(NH_2_-bdc)], through immersion of the crystalline framework in mIm linker solution. Investigation of the effect of aqueous solutions of both neutral, and equivalent pH to that of the linker solution, revealed that the crystallinity of MIL-68-NH_2_ was preserved without the presence of mIm linker. An alternative chemically-facilitated amorphisation was observed through the dehalogenation of [Cu^I^Cl(ttcH_3_)] (ttcH_3_ = trithiocyanuric acid), and partial removal of the ttcH_3_ linker to form [Cu^I^_1.8_(ttc)_0.6_(ttcH_3_)_0.4_].^110^

Elsewhere, Fe-BTC, [Fe(btc)], was prepared through post-synthetic metal-ion metathesis of CuZn-HKUST-1, [M3(btc)2], where complete substitution of both Cu^2+^ and Zn^2+^ with Fe^3+^ resulted in a loss of crystallinity.^111^ Metal-ion exchange also facilitated the collapse of MOF-5, [Zn_4_O(bdc)_3_] (bdc = 1,4-benzenedicarboxylate), to an amorphous material upon complete substitution of Zn with Fe.^112^ Interestingly, partial metal exchange with both Ni and Co resulted in a crystalline material isostructural with MOF-5.^112^

Whilst the formation of an amorphous material is possible with these methodologies, the process is energy intensive, the resultant structure is often unpredictable from the starting materials. Additionally, reduced porosity of the material after post-synthetic collapse has been observed, which limits industrial applications because the majority of applications rely on preserved porosity of the sample. To circumvent this, direct synthesis of these aMOF materials has been proposed.

### 2.2. Direct synthesis

**Fig. 5:**
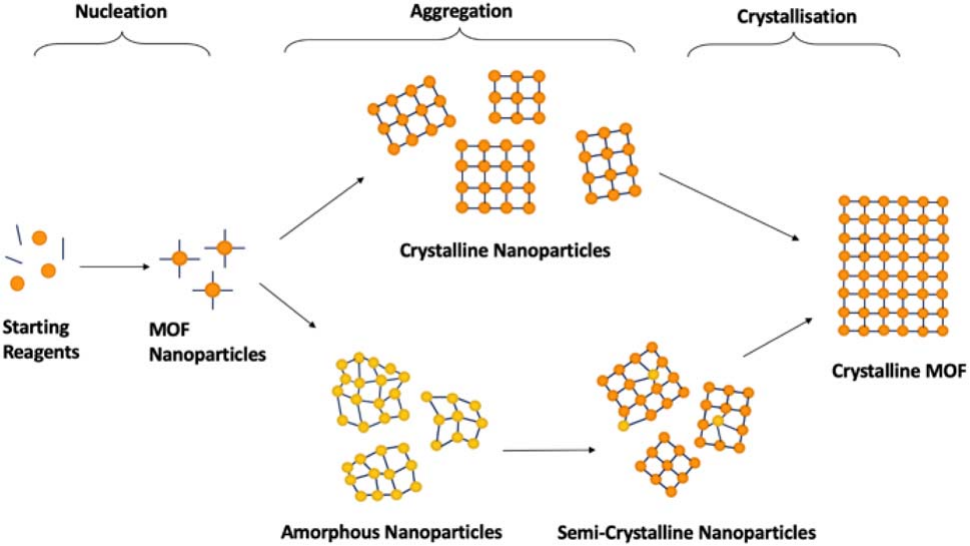
Schematic demonstrating 2D idealised potential structures present during the stages of crystalline MOF formation. Orange/yellow spheres represent metal clusters/SBUs, and blue lines represent organic linkers. The pathways are split into the three key stages, nucleation, aggregation and crystallisation.

Synthesis of crystalline MOFs commonly occurs through three key stages: nucleation, aggregation, also known as particle growth, and crystallisation (Fig. 5). However, there is still speculation as to the exact pathways followed.^51,69,113^ Nucleation refers to the formation of MOF nanoparticles from the starting materials through the self-assembly of isolated metal ions or pre-built inorganic building blocks with organic linkers. These are often described as SBUs joined with organic linkers but with no longer range connectivity, often defined as >7 Å.^51,52^ Subsequent assembly involves aggregation of these nanoparticles with extended connectivity, indicating either crystalline or amorphous particle growth. Finally, these organise to create structures with correlated order extending over the longer range during the crystallisation stage.^51,69,113^ Whilst investigation into the mechanism of progression has been performed for crystalline MOF synthesis, results have differed widely, despite minimal changes in synthetic conditions.^51,114,115^

The challenge with generalised a_s_MOF synthetic methods is the dependence of the mechanism of formation, and thus the parameters that govern it, on the MOF precursors. Incorrect understanding of how the MOF precursors affect the mechanism of formation can result in mixed-phase products, combining regions of crystallinity and disorder, or the absence of, or lack of predicted formation of, MOF material due to a lack of successful nucleation. Additionally, limited investigations have reported exactly where within crystalline synthesis sample ordering occurs, with variations noted depending on both synthetic procedure and starting reagents.^114,116–118^ Studies have noted that MOF nanoparticles can be nucleated both as crystalline and amorphous depending on reaction conditions, discussed later.^51,116^ Because of this, generalisation of MOF synthetic conditions is likely limited to specific combinations of SBUs and linkers which follow equivalent nucleation pathways. An additional challenge is the lack of available literature for different methodologies for synthesis of aMOFs. Synthesis of a_s_MOFs are often unreported, given their often-unwanted discovery during attempted cMOF synthesis. As such, synthetic tuning parameters to affect aMOF synthesis are almost totally underdeveloped. Excluding the recent publication by Zhang *et al.*, which outlined a methodology for the preparation of 22 amorphous MOFs, only 25 a_s_MOF structures prepared through direct synthesis, to the best of our knowledge, are known to have been reported at the time of writing this review.^119^ The methodology of these a_s_MOFs are outlined in Table 1. Synthetic methods of the comparative cMOF material were included only when outlined in the same publication.

**Table 1:** The composition and synthesis of reported a_s_MOFs and their relationship to their crystalline equivalents

Name	Synthetic conditions	Crystalline synthesis	Linker	Metal salt	Ref.
UiO-66	DMF, 90 °C, 1 month	DMF, 90 °C, 1 month	Terephthalic acid (H_2_BDC)	ZrCl_4_	120
UiO-66	DES-water, 100 °C, 5 h		Terephthalic acid (H_2_BDC)	ZrCl_4_	121
Zr-MOF(23)	DMF, 120 °C, 3 days		Ditopic clathrochelate complexes (^*t*^Bu substituent)	ZrCl_4_	122
Zr-MOF(24)	DMF, 120 °C, 3 days		Ditopic clathrochelate complexes (Br substituent)	ZrCl_4_	122
Zr-MOF(25)	DMF, 120 °C, 3 days		Tritopic	ZrCl_4_	122
			Clathrochelate complexes (Br substituent)		
Zr-MOF(26)	DMF, 120 °C, 3 days		Clathrochelate complexes (CO_2_H substituent)	ZrCl_4_	122
UiO-66-SO_3_H	DMF, acetic acid, 120 °C, 40 h	Introduction of aqueous alkali hydroxide solutions	2-Sulfoterephthalic acid (H_2_BDC-SO_3_H)	ZrCl_4_	123
CPP-1,-3,-5	DMF, acetic acid, 120 °C, 40 h		Biphenyl-4,4,-dicarboxylic acid (H_2_BPDC)	ZrCl_4_	124
aMOF-1	DMA, NH_3_·H_2_O, 90 °C, 3 days		2,6-Bis-(4-imidazole-1-yl-phenyl)-4-[4-(2*H*-tetrazol-5-yl)phenyl]-pyridine) (HBITP)	Co(NO_3_)_2_·6H_2_O	28
aMOF-2	DMA, NH_3_·H_2_O, 90 °C, 3 days		4-(4-(1*H*-Imidazole-1-yl)-phenyl)-2,6-bis(4-(2*H*-tetrazol-5-yl)phenyl)pyridine (H_2_IBTP)	Co(NO_3_)_2_·6H_2_O	28
Ti-*fum*	Anhydrous THF, ethanol, *m*-cresol, 85 °C (24 h) then 140 °C (8 h)		Fumaric acid (fum)	Ti_16_O_16_(OEt)_32_	96
ZIF-8	H_2_O, Go_*x*_ (enzyme), RT	Increased linker conc	Methylimidazole (mIm)	Zn(CH_3_CO_2_)_2_	125
Zn(ICA)-2	DMF, TEA, RT	Addition of hexane	Imidazole-2-carboxyaldehyde (ICA)	Zn(NO_3_)_2_·6H_2_O	126
Fe-BTC	Ethanol, RT		1,3,5-Benzenetricarboxylic acid (H_2_BTC)	Fe(NO_3_)_3_	127
MIL-89	H_2_O, ethanol, NaOH, 100 °C, <4 h	>4 h heating	*trans-trans*-Muconic acid (*tt*-MA)	Fe_3_O(CH_3_CO_2_)_6_·3H_2_O	128
MOF-74	Methanol, RT	aMOF-74 dispersed in H_2_O, heat (175 °C, 12 h)	2,5-Dihydroxyterephthalic acid (H_2_DHTA)	Co(CH_3_CO_2_)_2_·4H_2_O	129
In-IPA MOF	DMF, 140 °C, 20 minutes	HNO_3_, acetonitrile, imidazole, heat (80 °C 12h, 100 °C 24h)	Isophthalic acid (H_2_IPA)	In(NO_3_)_3_	130
Ni-pPD	Water, NH_4_OH, RT		*p*-Phenylenediamine (PPD)	Ni(NO_3_)_2_·6H_2_O	131
Ni^++^-MOFs	Water, DMF, microwave		1,3,5-Tribenzyl-1,3,5-triazine-2,4,6-(1*H*,3*H*,5*H*)-trione diphenyl-urea	Ni(CH_3_CO_2_)_2_·4H_2_O	132
Ni-MOF microsphere	DMF, glycol, 120 °C, 24 h		Terephthalic acid (H_2_BDC)	Ni(NO_3_)_2_	133
MOF microspheres	Adenine, DMF, H_2_O, 180 °C, 24 h		4,4′-Stilbenedicarboxylic acid (H_2_SDA)	Zn(CH_3_CO_2_)_2_·2H_2_O	134
NEU-3	2-MeTHF, pyridine, 100 °C, 24 h		*N*,*N*′-Pyromelliticdiimido-di-l-alanine (H_2_PMDA)	Zn(CH_3_CO_2_)_2_·2H_2_O	135
NEU-2	2-MeTHF, pyridine, 100 °C, 24 h		*N*,*N*′-Bis(glycinyl)-pyromellitic diimide (H_2_BPDI)	Fe(CH_3_CO_2_)_2_	61
NEU-4	2-MeTHF, pyridine, 100 °C, 24 h		*N*,*N*′-Pyromelliticdiimido-di-l-alanine (H_2_PMDA)	Fe(CH_3_CO_2_)_2_	135
NEU-5,-6,-7,-8	2-MeTHF, pyridine, 140 °C, 24 h		(FeTpyCOOH)(PF_6_)_2_ (5), (Ru(terpy*)_2_)(PF_6_)_2_ (6), (Ru(terpy*)_2_)(PF_6_)_2_ (7), (Ru(terpy*)_2_)(PF_6_)_2_ (8)	Zn(CH_3_CO_2_)_2_ (5,6)	136
				Fe(CH_3_CO_2_)_2_ (7)	
				Ti_4_(OCH_2_CH_3_)_16_ (8)	
a-Fe_1_Ni_2_(BDC-NH_2_)	DMF, acetonitrile, RT		Aminoterephthalic acid (H_2_BDC-NH_2_)	FeCl_2_·4H_2_O Ni(CH_3_CO_2_)_2_·4H_2_O	137
FeMn-MOF-74	DMF, ethanol, 120 °C for 12 h, 24 h, and 36 h	Increased temperature (135 °C, 150 °C)	2,5-Dihydroxy-1,4-benzenedicarboxylic acid (H_4_DHTA)	MnCl_2_, FeCl_2_	138
A_*x*_B_*y*_C_*z*_-NiFe MOFs	DMF, ethanol, water, TEA, sonication, RT		Terephthalic acid (H_2_BDC) (A), aminoterephthalic acid (H_2_BDC-NH_2_) (B), fluoroterephthalic acid (H_2_BDC-F) (C)	NiCl_2_·6H_2_O, FeCl_3_·6H_2_	139
MIL-37	DMF, 180 °C, 12 h	H_2_O solvent	3-Phosphonopropionic acid	Ni(NO_3_)_2_·6H_2_O, Fe(NO_3_)_3_·9H_2_O	140
NiFe-MOF-a/NF	DMF, 150 °C, 24 h	Increased temperature (170 °C)	1,3,5-Benzenetricarboxylic acid (H_2_BTC)	Fe(NO_3_)_3_·9H_2_O Ni(NO_3_)_2_·6H_2_O	141
MOF-M (Mg, Al, Fe, Co, Ni, Zn, Mn, Zr)	DMF, acetonitrile, 40 °C, 24 h		2,5-Dioxido-1,4-benzenedicarboxylic acid (DOBDC)	Zn(NO_3_)_2_·6H_2_O, Mg(NO_3_)_2_·6H_2_O Co(NO_3_)_2_·6H_2_O, FeCl_2_	142
				MnCl_2_	
				ZrCl_4_	
				Ni(NO_3_)_2_·6H_2_O Al(NO_3_)_3_·9H_2_O	

Analysis of these a_s_MOF syntheses revealed some consistency in synthetic methods across studies with constant metal salts, but varying linker structure and functionality. In the mixed-metal series of ‘NEU’ MOFs, a liquid–liquid interface synthesis was employed, with limited change in synthetic conditions, resulting in the formation of a_s_MOFs with a range of metal salts and linkers.^61,135,136,143^ This was achieved through controlled diffusion of reagents through an intermediate solvent, encouraging ligand-to-metal electron transfer. This is supported by the effective synthesis of A_*x*_B_*y*_C_*z*_-NiFe aMOFs, defined in Table 1, using a variety of terephthalic-acid based linkers without requiring changes to the synthetic conditions.^139^

These results suggest that development of a synthetic method for a particular amorphous MOF would likely translate to the formation of different aMOFs using similar synthetic conditions with variation in the functionality and structure of the linker and/or SBU. This provides confidence in the generality of research into amorphous MOFs, because even a few novel new aMOF synthesis strategies may lead to a growing library of new aMOFs. This has been demonstrated in a recent study utilising a modified Stöber method, typically used for the synthesis of aSiO_2_ colloids, to successfully synthesise a range of aMOF materials with differing SBUs and linkers.^119^ This highlights the potential for synthetic methodologies to be applied generally for the preparation of these disordered materials.

A more complicated problem is effectively modifying existing crystalline syntheses to produce a specific amorphous equivalent; however, it does at least provide a starting point for methodology development. In addition to the limited information on what controls the ordering during crystal growth of different MOF systems, there is substantial variation to crystalline synthetic methods that have been reported for the same MOF. For example, a multi-laboratory study investigated the reproducibility of crystalline Zr-porphyrin MOF synthesis, where the pure phase product was only prepared in one out of ten samples.^144^ Whilst this does make modification of the known method more challenging, it also highlights an area which a_s_MOFs could be beneficial, with the potential to be more synthetically reliable.

Several literature reports on MOF synthesis have focused on improving the crystallinity of a given MOF.^145–148^ However, information to the contrary, *i.e.*, understanding how to alter a crystalline synthesis to yield an amorphous product, is completely absent. The following section will therefore focus on potential methods and the theory behind directly synthesising aMOFs.

## 3. Synthetic factors controlling disorder

There are several methodologies commonly associated with the formation of cMOFs, including microwave-assisted, electrochemical, mechanochemical and sonochemical, with solvothermal being the most common.^66,149^ Employing a reverse logic to that used in methodologies that promote high crystallinity may well result in an amorphous phase.

Thermodynamic and kinetic factors governing MOF nucleation and crystallisation have been previously studied.^114,145^ Particularly, higher temperatures and longer reaction times yield more dense structures due to: (I) enthalpic penalties of larger frameworks without large degrees of non-covalent intra-pore stabilising forces, and (II) increasing entropic penalties with temperature. Conversely, lower temperatures and shorter reaction times tend to favour more porous products of higher energies.

### 3.1. Nucleation of MOF materials

Classical and non-classical nucleation pathways have been proposed for the formation of MOF materials.^114^ Classical nucleation defines nucleation as occurring through the coordination of atoms or molecules to form critical nuclei.^150^ These subsequently grow through the attachment of ‘monomer’ species. Any variation from this base-mechanism is termed non-classical nucleation.^151^ This is commonly seen through the presence of amorphous intermediates or pre-nucleation clusters.^152,153^ To identify how different parameters might affect the phase produced, an understanding of the mechanism of cMOF formation is required. Limited studies have focused on the early-stage molecular mechanism of cMOF formation, owing to complexities around their direct observation.

The majority of MOFs studied have been found to follow nucleation mechanisms which differ to that defined by classical nucleation theory. Classical theory has only been accurate for the description of a limited number of MOF species, with most studies focusing on HKUST-1.^154,155^ The majority of MOFs follow non-classical pathways, with pre-nucleation clusters (PCNs) being common.

One remarkable example can be seen in the case of UiO-66, [Zr_6_O_4_(OH)_4_(bdc)_6_], where *in situ* pair distribution function (PDF) analysis showed the presence of SBUs prior to MOF crystallisation, which are subsequently attached to each other through organic linkers.^117,156^ This was found to be consistent with observations of Hf-UiO-66, where precrystalline clusters were present prior to MOF formation.^157^ Interestingly, the small delay prior to the observation of crystalline material for the formation of UiO-66 has been attributed to the potential formation of an amorphous precursor, however there is little evidence as to its structure.^158^ Another example of this was observed through the formation of a three-fold paddlewheel, Mg_2_(Hcam)_3_, (Hcam = (+)-camphoric acid), prior to formation of the [Mg_2_(Hcam)_3_·3H_2_O]·NO_3_·MeCN MOF. The SBU subsequently assembles to form Mg_12_ cages, and then the extended MOF structure.^159^

An alternative to this method of nucleation is the formation of more general *meta*-stable intermediates, which have been found to be either crystalline or amorphous in nature. Formation of MOF-5 has been observed to form through cluster-based growth.^118,160–163^ One study identified the PNC within MOF-5 synthesis as Zn_5_(OH)_8_(NO_3_)_2_·2H_2_O nanoplatelets, which subsequently acted as nucleation sites for linker binding and MOF formation.^160^ Formation of MIL-53, [FeOH(bdc)], has also been observed to progress not through the pre-nucleation formation of the SBU, but through alternative *meta*-stable crystalline intermediates. Indeed, formation of MIL-53 involved the formation of an intermediate crystalline species structurally-related to MOF-235, [Fe_3_O(bdc)_3_(DMF)_3_][FeCl_4_].^164^

Interestingly, investigation into the formation of NH_2_-MIL-53-Al revealed the presence of an amorphous phase, prior to crystallisation into MOF-235 and subsequent structural rearrangement to MIL-53-Al.^165^ The interplay of both crystalline and amorphous intermediate species has also been observed in the formation of ZIF-8. Whilst literature has highlighted the rapid formation of crystalline Zn(2-mIm)_4_ clusters, an amorphous intermediate phase has also been noted, with similar structural features to that of the resultant crystalline material.^51,115,166–168^ This has been defined as a three-step nucleation pathway.^166^ The presence of an amorphous phase has also been observed in the synthesis of several other MOFs, including MIL-89, [Fe_3_O(CH_3_OH)_3_[O_2_C(CH)_4_CO_2_]_3_·Cl·(CH_3_OH)_6_], and ZIF-71, [Zn(dcim)_2_] (dcim = 4,5-dichloroimidazolate).^128,169^ The complexity and variation around MOF nucleation mechanisms is highlighted again through the investigation of ZIF-67, [Co(mIm)_2_], nucleation, where it was observed to progress through the formation of a ‘chemically diverse pool of metal–organic linker complexes’, rather than one defined PNC.^170^

These examples of the mechanisms by which MOFs nucleate highlight the challenges with generalisation across a range of materials, and indeed only presents a very simplified view. Further information about both the mechanisms and kinetics of these transformations can be found within the literature.^66,114,171^ Both classical and non-classical pathways to crystal growth have been shown for different cMOF materials, including monomer addition, amorphous-crystalline transitions, Ostwald ripening and aggregation mechanisms, however the extent to which these can be separated from the complex nucleation mechanisms outlined here is limited.^114^ An interesting example of the Ostwald ripening in MOFs is the multimetal [Zn_1−*x*_Co_*x*_(H_2_fipbb)] (H_2_fipbb = 4,4′-(hexafluoroisopropylidene)bis(benzoic acid)) which, after the nucleation of the needle-shape crystallites containing the Zn-SBU, ZnCo-SBU MOF shell grows around the Zn-SBU MOF core, generating a hole in the crystals.^172^

Although this highlights that classical theory is insufficient to describe both nucleation and growth mechanisms of MOF materials, some elements of classically-based models have been found to be applicable. Investigation into the formation of ZIF-8 revealed it followed the kinetic Avrami model closely.^115^ This study observed primary nucleation of MOF nanoparticles, followed by a fast increase in crystallinity during the growth stage. Indeed, Stock *et al.* noted the commonality of both the Avrami and Gualtieri models for description of the crystallisation kinetics of MOF formation.^66^ The Gualtieri model additionally notes the separation of nucleation and crystal growth stages, however information about early-stage crystallinity is often unpresented.^66,145^ In a recent study, this same model was found to be applicable for the description of the formation kinetics of Fe-BTC, despite the lack of order within the system. This implies that the nucleation stage of this model would support the formation of a disordered material.^173^

This suggests that, whilst mechanisms may differ across a range of MOFs, the kinetics which underpin the transformations, and thus what controls the rate of transformations, might be consistent. Reactant conditions, *i.e.* solvent, temperature, and concentration, have been observed to influence the relative rates of these stages.^115,118,155,174^ Nonetheless, methodology has been found to be applicable to a wide range of MOFs for control of both the nucleation and crystallisation stages, despite the likely variation in formation mechanism.^174^

Regardless of the exact model applicable to individual MOF syntheses, increased separation of the nucleation and crystal growth/crystallisation stages would allow for increased potential for the formation of a disordered material, reducing the likelihood of extended sample ordering. To this end, increasing the relative rate of nucleation compared to that of the crystal growth could allow for the isolation of an aMOF material, rather than a mixed-phase species. This would deplete the reactant solution of available MOF building blocks, and thus slow down any subsequent crystallisation taking place. It would also mean that any enthalpy-driven attachment of building blocks to the growing nuclei would take a back seat to kinetic-driven attachment, leading to disordered structures.

### 3.2. Specific synthetic methods

Increased MOF nanoparticle nucleation can often be observed through the formation of a MOF-gel synthesis, with a MOF-gel forming as a result of fast formation of an intramolecularly-bonded continuum of MOF nanoparticles within a reaction medium. Investigation into known solvothermal and sonochemical gel-based syntheses of cMOFs could therefore present insights into encouraging MOF nucleation. Reviews have been published exploring the formation and synthetic control of MOF gels, which could be constructive starting points for attempted aMOF synthesis.^56^ Alternatively, investigation into how the synthetic conditions control the defect concentration could inform methodology for the introduction of disorder into the system.

An alternative to adjusting cMOF-originated syntheses would be focus on aMOFs for which there are no crystalline equivalents, as this would indicate the crystalline phase is predominantly not thermodynamically (*meta*)stable. Previously, amorphous phases have been found to lie at significantly higher energy than more dense counterparts.^175^ This would allow for additional linkers and metal centres to be incorporated within aMOFs, to potentially unlock new beneficial properties not achievable with crystalline MOFs. Furthermore, the absence of a stable crystalline phase may allow the production of a truly pure topologically amorphous phase, which is not always the case with some of the materials reported in the literature (explored below).

Whilst solvothermal preparation of MOFs is common, an alternative is the utilisation of mechanosynthetic methods. This could present an interesting avenue for the formation of a_s_MOF materials. In mechanochemical synthesis, MOFs are formed *via* solid-state reactions induced by mechanical energy. This is typically facilitated through ball-milling or grinding, resulting in bond breakage and promoting of collisions between the reactant particles, leading to assembly of crystalline MOF materials.^176^ One of the advantages of this synthetic method is the near-complete reduction of solvent, commonly required for MOF synthesis.^176,177^ This not only allows for greener synthetic methods, but also removes the reliance on reactants' solubility, allowing for metal oxides to be employed as precursor materials.^176^ The exact mechanism of MOF formation in mechanochemical synthesis can vary, with several examples displaying direct formation of crystalline MOFs. Liquid assisted grinding (LAG) facilitated the direct formation of ZIF-8, shown from the diffusion-controlled kinetics of the reaction.^178^ This is consistent with the observations of UiO-66 mechanosynthesis, where diffusion-controlled kinetics were again noted, inconsistent with a precursor-based mechanism.^179^

In some cases, transient amorphous phases or metastable intermediates may still be involved, often under specific synthetic conditions. Indeed, whilst ion-LAG synthesis of crystalline ZIF-8 progressed directly, removal of the NH_4_NO_3_ LAG additive resulted in a change in kinetics, with synthesis likely progressing through the formation of an amorphous intermediate.^178^ The additive's effect on mechanistic pathway was neatly demonstrated with a systematic study on the mechanosynthesis of HKUST-1. *In situ* investigation revealed that varying the amount of liquid additive controlled whether cMOF formation occurred directly, or through alternative crystalline intermediates. Mechanochemical synthesis of MOF-74 was shown to progress through metastable crystalline intermediate phases, however the exact number of intermediates varies with publication, despite the same liquid additive being used.^180,181^ Due to the reaction being monitored using *in situ* powder X-ray diffractin (PXRD), it was unclear if amorphous intermediates were present in the early reaction stages.

*In situ* PXRD was also utilised to investigate the mechanosynthesis of ZIF-zni, [Zn(Im)_2_], confirming that mechanosynthesis proceeded through the formation of an amorphous ZIF intermediate.^182^ This was concluded through the fast loss of crystallinity, associated with the ZnO starting material, coupled with an increasing weight percentage attributed to an amorphous material.^182^ More generally, literature has suggested that the fast formation of MOF materials through LAG is likely due to the formation of a highly reactive amorphous intermediate phase.^183^ As the mechanism, and control of such, is still underdeveloped, implementing control of the resultant product is challenging. If the formation of stable amorphous intermediates can be encouraged, this could present an interesting avenue for the direct synthesis of a_s_MOF materials in the future.

### 3.3. Solvothermal synthesis

From known solvothermal methodologies, there are several potential parameters which can be controlled to not only encourage the formation of an amorphous material, but also prevent ordering to a crystalline MOF. These include reaction conditions, such as temperature and pressure, as well as the solvents, metal salts and modulators present.^70,184^ The potential effect of changing these parameters will be discussed in the following subsections.

#### 3.3.1. Temperature

The effect of controlling temperature on the resulting crystallinity of a MOF, as well as its crystal morphology, is well documented.^66,185^ Increasing the reaction time and temperature usually promotes the formation of the solvent-occupied crystalline phase in MOF synthesis, as this phase is generally the thermodynamically favoured product of the process. High temperatures facilitate solution saturation and the organisation of particles into a crystalline structure. Conversely, reducing the reaction temperature or heating time can alter the kinetics of MOF formation, favouring the kinetic product, often resulting in amorphous material.^52,70,149,186^ This phenomenon has been observed in various examples in the literature, explored below.^128,138,141^

Xu *et al.* determined that formation of cMOFs required the extended use of elevated temperatures, through the use of *in situ* PDF analysis.^117^ PDF data recorded in the early stages of the reaction revealed spectra consistent with the MOF SBU. Assembly of the extended cMOF structure was not noted until ∼15 minutes, where significant LRO was also observed (although some long-range order is suggested from as early as four minutes).^117^ This was supported by work on FeMn-MOF-74. Extended heating at 120 °C resulted in the formation of a topologically amorphous MOF material, while heating at 135 °C resulted in sharp Bragg peaks appearing in the X-ray diffraction (XRD) pattern, indicating crystallinity.^91^ Investigation of MIL-89 formation noted, again, the presence of an amorphous material containing the defined SBU at early stages of the solvothermal synthesis. Continued heating of the reaction media resulted in the formation of the crystalline MOF, as with above.^128^

One of the routes to obtain an a_s_MOF could therefore be through performing the crystalline synthesis at reduced or room temperature, or decreasing the reaction time.^120^ Indeed, there are several reports of a_s_MOFs prepared at room temperature, though not all have crystalline equivalents for comparison of experimental conditions.^139,187^ Whilst this may imply a straightforward methodology, the insolubility and stability of the starting materials would likely render this unpredictable when using different reagents. This unpredictability could lead to low rates of nucleation, restricted formation of MOF nanoparticles, and the possible preferential hydroxylation of the metal salt in solution, resulting in the formation of metal oxides. Additionally, room temperature synthesis is no guarantee of formation of an aMOF. For instance, during a room temperature synthesis, particles of ZIF-8 nucleating were observed to nucleate and possess a crystalline structure when examined using liquid cell transmission electron microscopy (TEM).^116^ This was in contrast to alternative ZIF-8 synthetic methods, where an amorphous-crystalline transformation was noted, through the use of cryo-TEM, in the early-stages of the reaction.^51^ Whilst both reactions were conducted at room temperature, variations in solvents and the ratio of starting reagents were noted, indicating that controlling the reaction mechanism does not rely solely on the reaction temperature.

An alternative modification would be the application of lower temperatures over an extended period of time, however this can result in the formation of a mixed-phase material.^28^ Low temperature synthesis of a Ti-based polymeric MOF, comprised of both bidentate *syn*-(Me, Me)-bimane and monodentate *anti*-(Me, Me)-bimane ((Me, Me)-bimane = C_10_H_12_N_2_O_2_), was achieved through utilisation of microwave radiation. This was revealed to form a partially crystalline material, rather than the equivalent cMOF, which was the original focus of the research.^188^ Although the material is reported as amorphous, XRD spectra revealed the presence of limited Bragg peaks, with analysis suggesting the amorphous phase dominated ∼55% of the sample. No distinction was made about the connectivity of the crystalline and amorphous phases. Another example involved the solvothermal preparation of a_s_UiO-66 at 90 °C, a comparatively low temperature to cMOF synthesis, resulted in the simultaneous formation of both the crystalline and amorphous material, which could subsequently be separated, defined above as a partially crystalline material.^120^ Various cMOFs are also synthesised at temperatures at or below room temperature.^115,127,189,190^ This demonstrates the challenge with forming pure amorphous phases using only lower temperatures and longer timeframes.

#### 3.3.2. Modulators

Traditionally, modulators are deprotonated monodentate acidic ligands, based on *e.g.* acetate, benzoate and formate. Control of the resulting phase is realised by the competitive bonding of the modulators with the linkers, slowing down bond formation and allowing time for the structure to order.^118,191^

Several crystalline MOF syntheses require the use of a modulator to form a highly crystalline material or to control the phase that forms.^9,191–193^ This was observed for a UiO-66-type MOF, which noted an increase in crystallinity upon inclusion of an amino-acid based modulator into the synthesis.^194^ An equivalent increase in crystallinity was observed with Zr-*fum*, [Zr_6_O_4_(OH)_4_(fumarate)_6_], upon addition of either formic acid or benzoic acid modulators.^195^ Literature suggests this is likely due to the coordination of the modulator to the SBU, reducing the rate of crystallisation, and resulting in the formation of a more ordered MOF.^195^ Considering the link between modulator selection and resulting crystallinity, one might hypothesise that removal of the modulator from a known synthetic procedure could produce an amorphous MOF.

**Fig. 6:**
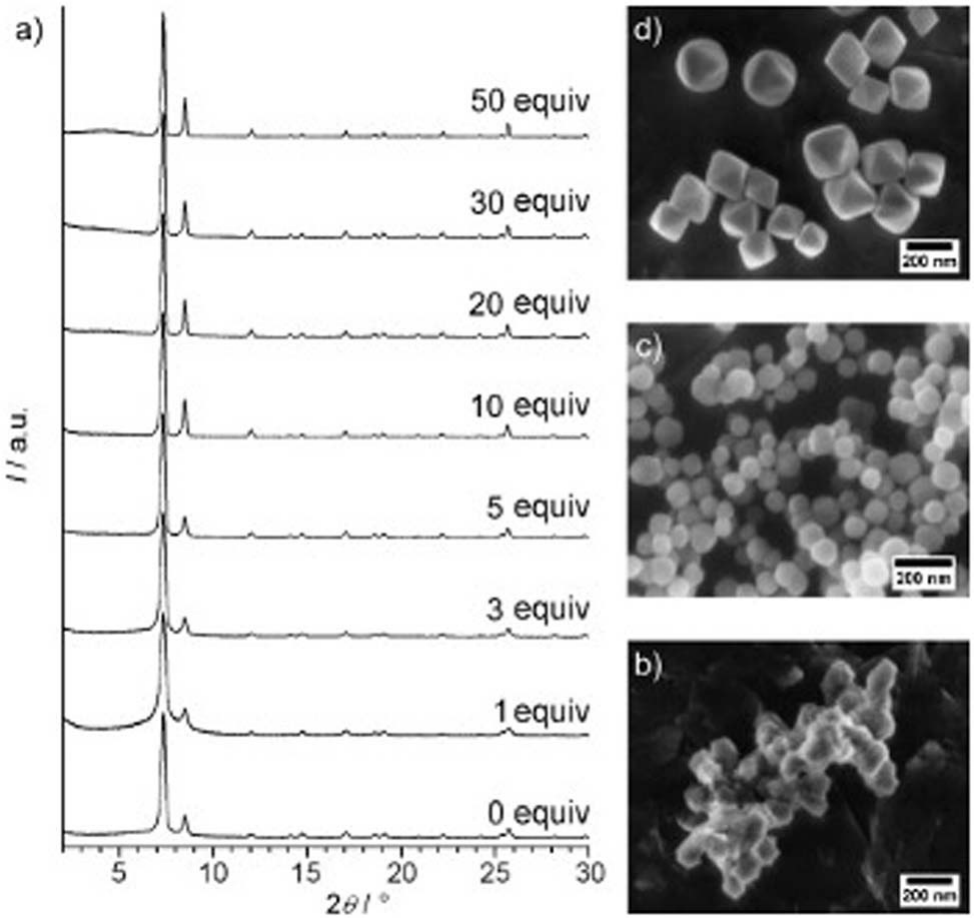
(a) Powder XRD patterns of Zr-bdc MOFs prepared with different amounts of benzoic acid (given as equivalents with respect to ZrCl_4_) as the modulator. SEM images of Zr-bdc MOFs synthesised in the presence of (b) 0, (c) 10, and (d) 30 equivalents of benzoic acid are also given. Reproduced from Schaate *et al.* with permissions from Wiley 2011.^191^

For Zr-*fum*, removal of the formic acid modulator resulted in the formation of a highly disordered structure, confirmed through PXRD.^196^ This was also observed upon removal of a benzoic acid modulator from the synthetic method when preparing the same MOF.^197^ This contrasts with what was observed for UiO-66, where removal of the benzoic acid modulator resulted in the formation of a nanocrystalline, rather than an amorphous material, suggested from the position of the broad peaks seen from PXRD (Fig. 6).^191^ This again highlights challenges with classification of sample order from PXRD alone. In another example, direct synthesis of aUiO-66 was achieved when the modulator was removed from the synthetic method, in addition to decreasing the reaction temperature and removing the autogenous pressure from the system.^120^ Interestingly, the original synthesis of crystalline UiO-66 in this experiment did not require a modulator but did utilise higher temperatures than the modulated synthesis.^198^ This highlights the importance of the interplay of different synthetic parameters to control the crystallinity of a MOF system.

#### 3.3.3. Solvents

The effect of the solvent has been widely investigated for the control of the synthesis of both cMOFs and a_s_MOFs.^199,200^ Theoretically, addition of a solvent that the MOF precursors are insoluble in would limit the formation of MOF nanoparticles. Whilst this would likely result in a decreased yield of the reaction, it would also decrease the rate of the subsequent aggregation and ordering stages of cMOF formation, likely producing an amorphous MOF.

Within the literature, most amorphous MOF syntheses have been conducted with DMF as the solvent. This is likely because of the commonality of this solvent in crystalline MOF synthetic methods, where many MOF precursors are insoluble in other solvents. High solubility of the precursors are required for cMOF synthesis, but this is not necessarily the case for formation of an a_s_MOF. Insolubility of reagents could be utilised to limit MOF nanoparticle growth/ordering. This could allow for a wider range of solvents, as well as potentially greener alternatives, to be utilised.

The importance of solvent interactions on the stability of the crystalline phase was noted through the amorphous-crystalline transition of an infinite coordination polymer upon immersion in a MeOH solution.^201^ This was concluded to be caused by the coordination of MeOH to the metal ions.^201^ Given that direct crystalline synthesis was noted to occur when performed in MeOH, these post-synthetic transitions could inform on the solvents which stabilise equivalent species, and *vice versa*.

Water has been shown to be a useful alternative solvent for a_s_MOF formation (Table 1). Motivation for the selection of water as a solvent stems from the theory that strong interactions between the water molecules and the metal sites of the MOF increase the disorder of the system.^68^ The strong coordination of H_2_O to the metal centres results in the build-up of a critical concentration of linker defects, resulting in an inability to form an ordered structure. This is consistent with the known instability of crystalline MOFs in water, often causing structural collapse.^202^ A water-based synthesis of a_s_ZIF-8 has been noted in the literature, despite the application of elevated temperatures.^51^ Extended application of heat did, however, result in subsequent ordering of the sample to form a crystalline material. The existence of this early-stage amorphous material was important, as alternative solvents were seen to produce a crystalline material from the offset.^116^

In contrast, attempts to synthesise [Cu_3_(btc)_2_] in water resulted in the formation of a collapsed MOF network, with Bragg peaks present, but inconsistent with the known crystalline phase. This phase was only produced once >30% v/vol ethanol was added.^203^ At concentrations lower than this, it was concluded that the linkers had not coordinated to the metal centres.^203^ Interestingly, for the synthesis of aMIL-37,^142^ [Fe[(OH)(O_3_P(CH_2_)_2_CO_2_H)]·H_2_O], and aMOF-74,^134^ exchanging DMF and methanol respectively for H_2_O resulted in the formation of the crystalline material, rather than amorphous. It should be noted that, in addition to introduction of water into the system, the formation of crystalline MOF-74 also required heating, whereas the amorphous synthesis was performed at room temperature.^129^

As an alternative to traditional solvents, deep eutectic solvents (DES), a class of ionic liquid composed of a mixture of Lewis/Brønsted acids and bases, have begun to be explored for the preparation of a_s_MOFs. aUiO-66 was directly synthesised at 100 °C utilising DES to provide hydrogen bond donors and acceptors to stabilise the formation of a highly defective system.^121^ It was noted that the variation of acid and base comprising each of the DES affected the defect concentration within the aMOF systems, likely due to the strength of the interactions that occurred.^121^

#### 3.3.4. Reagent concentration and ratio

In addition to solvent effects, the metal/ligand ratio, as well as the concentration of these precursors in the solution, are also important factors for controlling the crystallinity of MOFs. For example, a study on the synthesis of ZIF-8 encapsulated enzymes showed that a low ligand-to-metal ratio resulted in irregular coordination of the linker, which decreased the crystallinity of the MOF to produce a topologically amorphous material.^125^ This was unexpected, as the rate of nucleation is often linked to the supersaturation of the reactants.^204^ Since ZIF-8 has been observed to nucleate *via* the formation of an amorphous intermediate phase, separation of this amorphous precursor from crystal growth would have been expected to yield a topologically amorphous product.^51^

The ligand to metal ratio has also been shown as a way of controlling the structure, rather than just the crystallinity, for Co-based MOFs with bis(imidazole) ligands.^205^ Through an investigation of core–shell MOF structures, Wu *et al.* observed that modification of metal–metal ratio in the reaction mixture affected the crystallinity of the product.^206^ Upon increase of the relative proportion of Fe^3+^:Mn^2+^ above 66%, a crystalline material no longer formed, instead resulting in the formation of amorphous clusters.^206^

An alternative methodology that could be applied to the direct synthesis of amorphous MOFs utilises competitive linker binding, something currently employed within *de novo* defect engineering of crystalline materials, discussed above.

#### 3.3.5. pH

Another important reaction parameter affecting the crystallinity of a MOF is pH. Bauer *et al.* also investigated the effect that the reaction solution pH has on crystallinity for Fe-based MOFs, namely Fe-MIL-88-NH_2_, Fe_3_O(solv)_3_X(NH_2_-bdc)_3_·msolv (X = Cl^−^, Br^−^; solv = H_2_O, DMF, CH_3_OH, CH_3_CN), MIL-53-NH_2_, and Fe-MIL-101-NH_2_, Fe_3_O(solv)_3_Cl(NH_2_-bdc)_3_.^200^ It was found that a combination of highly acidic, aqueous conditions and low temperatures tended to produce aMOF materials.^200^ On the other hand, using the same reagents but increasing the temperature and using basic conditions also produced an a_s_MOF.^200^ This highlights the challenge in understanding the effect individual parameters have on the disorder of the MOF, and indicates that a combination of parameters would likely need to be considered.

In addition to this, pH-responsive amorphous-crystalline phase transitions from UiO-66-SO_3_H to UiO-66-SO_3_M (M = Li, Na, K) have been observed upon the addition of alkali hydroxide solutions.^123^ This pH-responsive behaviour was suggested to be caused by strong hydrogen bonds breaking, allowing the disordered framework to expand, and subsequently reorder.^123^ Interestingly, immersion of crystalline UiO-66-NO_2_ in a NaOH solution resulted in a loss of order because of increased linker defects.^207^ The complexity surrounding pH control of crystallinity was highlighted further when, upon the incorporation of DES with different pH's for aUiO-66 synthesis, no change in crystallinity was observed.^121^ This contrasts with Zr-Tyr, (Tyr = tyrosine), MOFs where basic conditions revealed the formation of amorphous/partially crystalline MOF material.^208^

A Stöber methodology, found to be applicable with a range of metals and linkers, incorporated the addition of a base to the reaction mixture.^119^ Through introduction of a base, in this case TEA (triethylamine), the acidic linker can be ‘activated’, or deprotonated, resulting in faster ligand attack.^119^ This provides kinetic control to the formation of MOF materials, as the concentration of base was found to affect the rate of nucleation.^119^ Whilst direct addition of TEA resulted in limited controllability of aMOF growth, gas-phase diffusion allowed for continued formation of deprotonated linker, which is key for controlled nucleation and growth.^119^ This methodology was applicable to a variety of both the metal clusters and organic linkers, which highlights the potential for generalised synthetic procedures for the synthesis of a_s_MOFs.

## 4. Applications

**Fig. 7:**
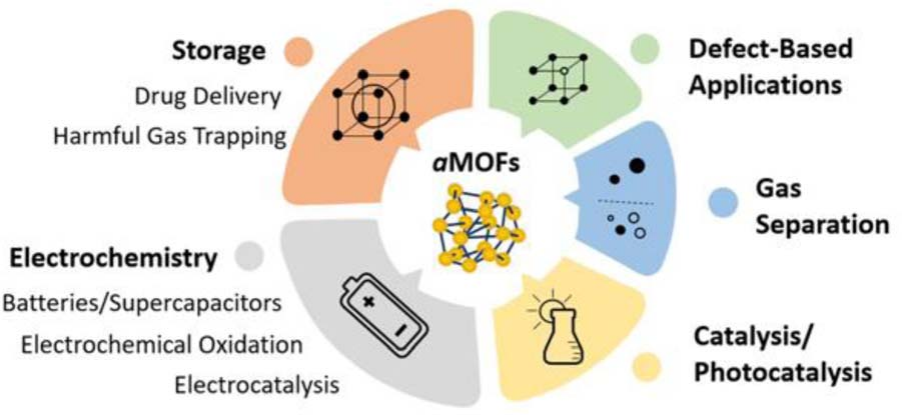
Applications of aMOFs in the literature.

There are several applications that have been explored for both directly synthesised and post-synthetically amorphised aMOFs (Fig. 7). One of the most common applications of aMOFs revolves around collapsing a porous crystalline structure around a guest. One example of this was the trapping of iodine, through amorphisation of crystalline ZIF-8 around the I_2_ molecules, utilising both ball-milling and pressure.^76,82^ This concept can be utilised for the long-term storage of harmful materials.

This trapping behaviour was additionally utilised for the controlled release of drug molecules. Encapsulation of a drug within the pores of UiO-66 through ball-milled amorphisation showed an increase in release time from 2 to 30 days, relative to the crystalline material.^64^ This behaviour was mimicked for a range of Zr-based MOFs with varying linkers.^209^ Additionally, thermal amorphisation of CAU-7, [Bi(btb)] (btb = 1,3,5-benzenetrisbenzoate), has also been observed to increase the release time of several drug species.^210^ Whilst these examples relied on post-synthetic amorphisation methodologies, this trapping behaviour could also be applied to a_s_MOFs, but only if the synthesis can direct network construction around the targeted species.

Amorphous MOFs have also been investigated for use in electrochemical applications such as supercapacitors and secondary ion batteries, and in electrochemical water oxidation reactions.^54,211,212^ A perspective has been recently published outlining the potential future benefits of aMOFs within electrochemistry, because of the potential for improved close packing and orbital overlap for interfacial charge transfer in aMOFs.^213^

As mentioned in the introduction, one of the desirable intrinsic properties of amorphous MOF systems is their capacity to support high defect concentrations, compared to their crystalline equivalents. Interestingly, the idea of defects within a directly synthesised amorphous structure presents a challenge. As opposed to crystalline materials, where an equivalent structure exists with no, or limited, defects, there are no non-defective equivalents of disordered materials. In the case of amorphous materials, defects seem to be localised to missing linker or missing cluster defects, which could instead be referred to as coordinatively unsaturated sites. Whilst this terminology may make more rational chemical sense, the term ‘defective’ will likely still be utilised for easy comparison to existing systems.

It is the presence of a high concentration of these unsaturated sites within aMOF systems, regardless of their method of amorphisation, that can change the materials' properties. These defects equate to active sites, which are particularly beneficial for both catalysis and adsorption, providing active sites for both.^59,214–216^ This was demonstrated elegantly with UiO-66, where a high concentration of defects was thought to increase both the pore size and the BET surface area, leading to an increased sorption capacity of N_2(g)_.^45,217^ This is supported through investigation of an Ir-based MOF system, where increased defect concentration within the crystalline material resulted in a higher adsorption capacity compared to that seen within the non-defective parent.^50^ For the crystalline analogue, increasing the defect concentration past a certain point produced an unstable structure, which collapsed to an amorphous phase.^50^ Additionally, the formation of large-scale mesoporous defect structures, created by increased defect concentrations, have improved mass-transport pathways which can be beneficial for gas adsorption and water purification.^218^

**Fig. 8:**
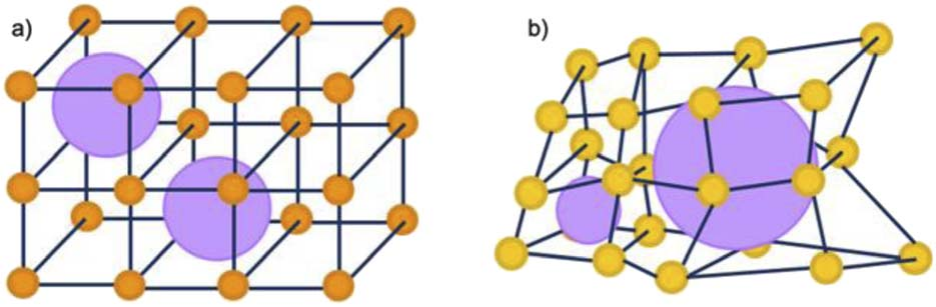
Schematic representation of the potential porosity of 2D MOFs that are (a) crystalline and (b) amorphous in nature. Orange/yellow spheres represent metal clusters/SBUs, and blue lines represent organic linkers, purple spheres represent potential porosity.

Whilst a high defect concentration present in these amorphous phases could have a generalised improvement for certain applications, the activity of these materials is often dependant on their structure and porosity, which is often compromised through post-synthetic amorphisation. Directly synthesised aMOFs present the benefit of a preserved, albeit modified, extrinsic porous structure. This can result not only in an increase in the number of catalytic sites, but also a potential for accessing previously inaccessible volume through the inclusion of missing linker defects, consistent with existing research in the area, idealised in Fig. 8.^31,59,69,219,220^ Whilst structural collapse often leads to a change in the intrinsic microporosity, the irregularity of the structure often allows for the presence of irregular mesoporous structures within the material. This modifies the extrinsic porosity of the material and potentially allowing for previously inaccessible porosity to be utilised.

A similar effect was observed in well-known crystalline-amorphous analogues, MIL-100 (crystalline) and Fe-BTC (amorphous/disordered). Despite the lower BET surface area of Fe-BTC compared to MIL-100, the latter displayed improved proficiency for the gaseous separation of propene and propane compared to the crystalline analogue.^60,221^ The improvement in separation capability of Fe-BTC was suggested to arise from linker defects in the disordered structure, which produced a higher concentration of metal active sites. This reasoning also explains the increased specific capacitance of a_s_UiO-66 compared to its crystalline equivalent.^120^ Additionally, despite the reduced porosity of a_s_UiO-66, it displayed trapping behaviour upon adsorption of N_2_, something not observed with the crystalline equivalent, with potential application in harmful gas storage.^120^

An alternative synthetic method for a_s_UiO-66 revealed an increase in sorption capacity compared to the crystalline material, again for N_2_ adsorption.^121^ Co-based a_s_MOFs have also been seen to display higher adsorption capacities for iodine and organic dyes compared to several crystalline MOFs, again showing the potential for porosity within these directly synthesised aMOF systems.^28^ a_s_ZIF-8 observed a mesoporous structure, which conferred up to 20 times the catalytic activity to the encapsulated glucose oxidase compared to the crystalline equivalent, again, likely due to the increased defect concentration.^125^ aMIL-68-NH_2_ was prepared post-synthetically through competitive ligand binding, allowing for the preservation of the parent porous structure.^62^ This meso- and microporous MOF demonstrated increased activity for both photo- and electrocatalysis, likely because of the missing linker defects present in the structure.^62^ This highlights the general advantage of these amorphous MOF systems, as well as the large scope of the applications.

The method of solvent removal has been shown to be vital to maintain the porosity of these a_s_MOFs. For example, with the formation of aerogels through supercritical CO_2_ drying noting improved porosity compared to air-dried samples.^127^ Templating has been utilised in crystalline MOF synthesis to encourage the formation of hierarchical structures with improved accessible mesoporosity.^222,223^ Application of the same structure directing agents (SDA) within a_s_MOF synthesis would potentially allow for forced inclusion of mesopores within the disordered structure. Removal of the templating agents could, in theory, increase the porosity in an equivalent way to the crystalline material. Since amorphous structures possesses less rigidity than that of the crystalline equivalents, the methodology would have to be altered so that the SDA could be removed without collapsing the induced extrinsic porosity of the material.

An additional advantage currently observed with aMOFs formed by post-synthetic collapse of cMOFs is the improved mechanical stability compared to their crystalline analogues, which could allow for increased processability of these materials.^56^ The general increase in hardness observed has been explained by the lack of defined grain boundaries or defects within the aMOF structure, resulting in no clear points of structural weakness or slip.^6^ This is likely a property which would be conferred to a_s_MOF materials. This would suggest application of additional stress to the aMOF material would likely have reduced effect on the structure or properties, compared to that of the crystalline equivalent. This could result in easier formation of MOF thin films, where cMOF thin films are used in optical and chemical sensors, catalysis and electrochemistry.^224–228^ The potential for increased exposed defective sites within aMOF thin films could allow for increased activity with respect to these applications, with defect engineering of cMOF thin films noted to show interest for tuning redox conductivity.^229^

Additionally, gel-based preparation of a_s_MOFs could result in the formation of monolith structures, observed currently with crystalline MOFs. Monolith structures would have higher mechanical stability, in addition to improved gas sorption capabilities, which expands the application potential of these materials further.^56,219,230^ The potential for improved gas sorption has been observed with the direct synthesis of Ti-*fum*, and amorphous MOF monolith.^96^ Ti-*fum* was prepared through the slow evaporation of solvent after MOF synthesis was performed, producing an amorphous material with a high BET surface area.^96^

## 5. Challenges with characterisation

Several key challenges present themselves during the structural characterisation of amorphous MOFs. Differentiation of the states defined in Fig. 1, and thus identification of a material which is truly topologically amorphous is often challenging. In particular, differentiating samples containing nanocrystallinity compared to those possessing extreme disorder has inherent difficulties. The characterisation of an aMOF is further complicated by the experimental difficulty of forming a pure phase, highlighted by the frequency with which poorly crystalline or partially crystalline samples are reported within the literature.^231,232^ The second issue stems from aMOFs' inherent lack of structural periodicity, limiting the information available from several key techniques. This is especially prevalent with X-ray diffraction, with the majority of structural characterisation possible from this technique focusing on the presence of either a single crystal or a unit cell. Advancing current characterisation techniques will improve understanding on how the short-range order of an amorphous material affects the properties, furthering the potential for rational design of aMOFs.

### 5.1. Confirming lack of long-range order

One of the most common techniques used for the identification of an amorphous material is powder X-ray diffraction (PXRD). Whilst highly crystalline materials produce sharp, well-defined Bragg peaks, amorphous materials only exhibit diffuse scattering.^6,108,233,234^ Despite the limited structural information obtained from PXRD, it is routinely used to track the formation of an aMOF upon application of heat, pressure or stress to a cMOF through both *ex*- and *in situ* studies. With these studies, the samples are generally concluded to be fully amorphous when the Bragg peaks present within the starting material have broadened significantly, becoming almost indistinguishable from the baseline.^235^

*In situ* studies, performed with a variety of experimental conditions, have been vital in identifying the conditions at which crystalline-amorphous structural transitions occur.^75,236^ Recrystallisation can also be observed, which might give an indication of the mechanism of structural collapse, and whether it involves a reconstructive transition.^71,78^ PXRD has also been utilised to draw comparisons between aMOFs prepared using a variety of post-synthetic methods, suggesting either the formation of a fully amorphous phase,^75,81^ or the potential preservation of some partial crystallinity for differing methodologies.^232^ PXRD has also been used to confirm the direct formation of amorphous MOFs. The successful preparation of a_s_UiO-66 was confirmed, in addition to other techniques, by PXRD where three broad diffuse scattering features were present in the pattern, confirming a lack of LRO.^120^

**Fig. 9:**
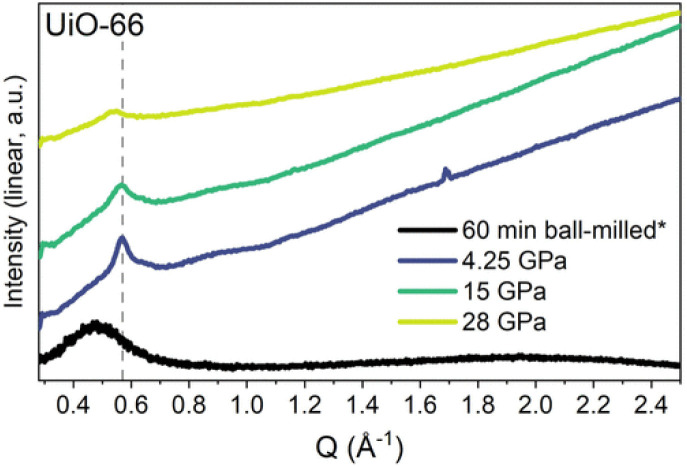
PXRD data for UiO-66 after ball-milling for 60 minutes and at the maximum pressure of each *in situ* hydrostatic compression. Data were taken using either a lab source (asterisk) or a synchrotron. Data were normalised using the most intense Bragg peak (111 reflection) under ambient conditions. Figure reproduced with permission from Robertson *et al.*^232^ Copyright 2023 American Chemical Society.

Nanocrystalline materials exhibit near-equivalent broad PXRD peaks to those found in amorphous samples. This is a result of the inverse relationship between peak full-width half maximum and the particle size, defined by the Scherer equation.^237,238^ This has made differentiation between nanocrystalline and truly amorphous samples difficult using only PXRD, despite its current place as the primary characterisation technique applied. This has likely contributed to uncertainty within the literature, where materials have been misidentified as fully topologically amorphous. Clues are often provided where the broad peaks of low intensity are centred on equivalent peaks in the crystalline sample, however this is only an indication and a qualitative measure.^146^ Several studies have analysed the effect of different modulators on the synthesis of UiO-66, but challenges arise when classifying the resulting product by PXRD as nanocrystalline, poorly crystalline or topologically amorphous. Similarly, structural investigation of Fe-BTC by PXRD revealed the presence of broad Bragg peaks, however additional techniques were required to distinguish if the material possessed nanocrystallinity, disorder or a combination of the two.^221^ To further illustrate this challenge, different methods of post-synthetic amorphisation of the same MOF was observed to produce differing broad PXRD spectra. Whilst Zr-MOFs exposed to high pressure exhibited broad Bragg peaks (Fig. 9), defined as a quasi-amorphous spectrum, the pattern was inconsistent with that of a ball-milled amorphous MOF of the same crystalline precursor, suggesting that the compressed material could be partially crystalline in nature.^232^ Additionally, upon decompression, some Bragg peak intensity was recovered, again suggesting that the apparent level of disorder of the material cannot be concluded solely from the PXRD spectra.^232^

One of the key techniques for analysis of short-range, or local, order is pair distribution function (PDF) analysis. PDF is a total scattering technique which simultaneously analyses both Bragg and diffuse scattering, giving the probability of atom–atom correlations of distance *r*.^239^ For amorphous samples, peak intensity drops significantly at higher *r* values, indicating an absence of correlated long-range order, whilst short range order of a material is maintained. Whilst the majority of the literature available utilises high-energy X-rays to collect PDF data, both electron and neutron PDF are becoming increasingly common.^231,240,241^ Since certain elements have increased sensitivity to different methods of collection, combining these could potentially provide a more complete view of the short-range order and thus the mechanism of collapse of the correlated structure.^242^

**Fig. 10:**
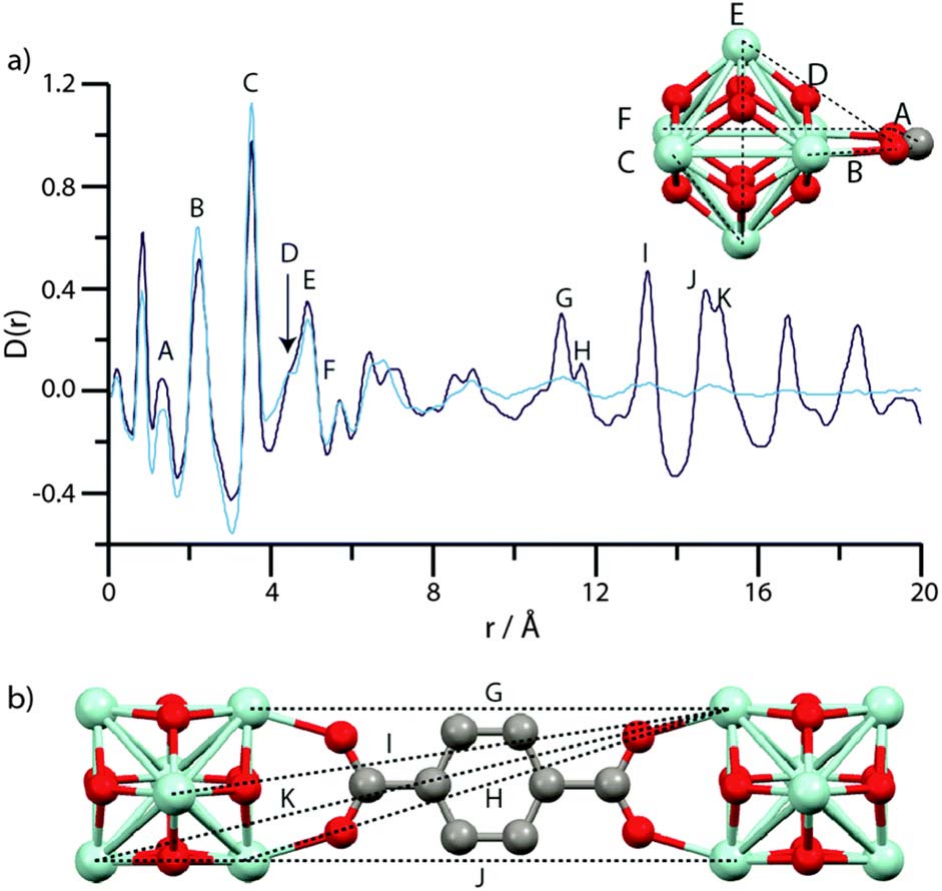
(a) PDF data for UiO-66 (dark blue) and a_m_UiO-66 (light blue). (A–F) labels of peaks below 8 Å correspond to the indicated correlations in the Zr_6_O_4_(OH)_4_ cluster (inset). (b) Two Zr_6_O_4_(OH)_4_ units linked by a bdc ligand, and some of the significant distances corresponding to the longer r features (G–K) in (a). Zr – light blue, O – red, C – gray, H – omitted. Figure reproduced from Bennett *et al.* with permission from the PCCP Owner Societies.^86^

Comparing PDFs of crystalline MIL-100 and disordered Fe-BTC showed similarities below 10 Å, indicative of comparative short-range order.^221^ The lack of any noteworthy long-range order, but defined medium-range order, was consistent with their classification of Fe-BTC as nanocomposite, nanocrystalline-amorphous structures, based on trimer and tetramer building units present within the crystalline material.^221^ Elsewhere, the resultant structure of a_m_UiO-66 was compared to the crystalline parent equivalent utilising PDF (Fig. 10). From this, both complete loss of LRO was noted, through lack of high-intensity correlations at significant distance, as well as consistency in the SRO, suggesting SBU is preserved upon amorphisation. Whilst this is not a directly synthesised amorphous sample, it highlights the information that could be gained from the technique.

Additionally, progressive amorphisation of materials can be monitored by PDF through the decrease in intensity of the LRO-based correlations. Investigation of post-synthetic amorphisation revealed both the complete loss of correlated LRO, but also either preservation or change of the SRO of the system depending on both the MOF and the methodology.^83,86^ Investigation into both the thermal and mechanochemical amorphisation of several ZIF species revealed collapse to structures with equivalent SRO, regardless of methodology.^71,87^ Utilising this technique, insights can be made into the mechanism of collapse, and the potential predictability of the resultant structure. Application of this technique to directly synthesised materials would potentially allow for prediction of the properties relative to the crystalline material based on any equivalent structural features.

The primary challenge associated with PDF is the need for accurate chemical compositions, where a combination of techniques, such as nuclear magnetic resonance,^33,243–245^ elemental analysis^221,246^ and inductive-coupled plasma mass spectroscopy^243,247^ should be used in tandem. In addition to this, thermogravimetric analysis (TGA) can be utilised to give the defect concentration of the MOF, again indicating the composition of the material.^44,248–250^ Importantly, X-ray or neutron PDF informs only on the bulk SRO of the material. There are several techniques, explored below, which investigate atom-specific local structure and coordination. This would be particularly beneficial for directly synthesised aMOFs, to potentially highlight regions of increased order within the sample.

To identify localised crystalline or amorphous phases, as well as structural defects, both normal and high-resolution transmission electron microscopy (TEM) have been utilised.^251,252^ In these techniques, Bragg spots in the diffraction pattern are indicative of a highly crystalline sample, resulting from constructive interference of the elastically scattered electrons. Differentiation between a nanocrystalline and a truly amorphous sample is therefore possible, with the nanocrystalline sample still displaying sharp spots regardless of crystallite size.^253^ This is compared to the diffuse scattering present with a lack of long-range order. For example, HR-TEM was recently used to investigate the structure of nanocrystalline UiO-66, where PXRD proved ineffective.^254^ HR-STEM was applied, in addition to PXRD, for the investigation of MIL-100 and Fe-BTC, specifically to determine if the Fe-BTC was nanocrystalline or amorphous.^221^ Whilst crystalline lattice fringes were present in both samples, Fe-BTC observed these over a smaller field of view, consistent with a partially crystalline sample.^221^

**Fig. 11:**
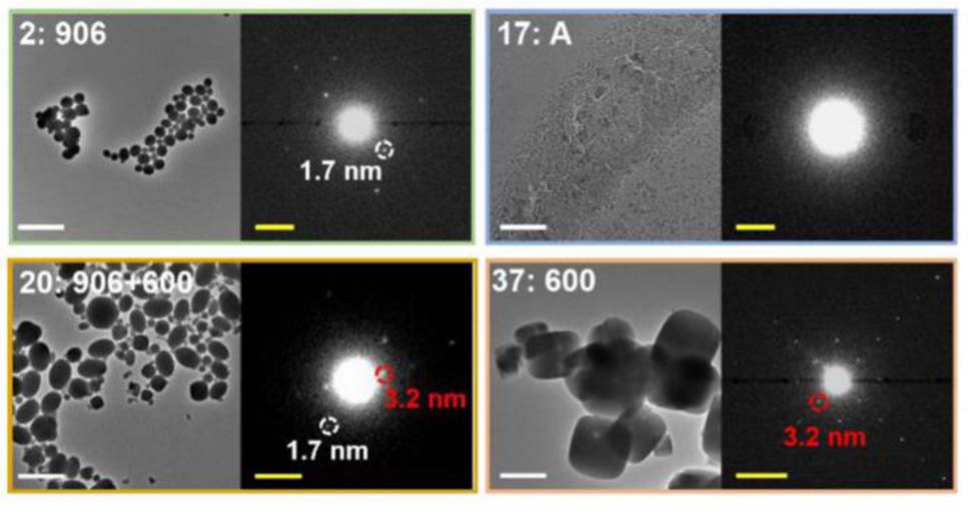
Zoom-ins with enlarged diffraction patterns for selected samples from an array. The observed phases are donated as A (amorphous product), 906 (NU-906), or 600 (NU-600). White scale bars = 2 μm. Yellow scale bars = 0.5 nm^−1^. Reprinted (adapted) with permission from Gong *et al.*^255^ Copyright 2022 American Chemical Society.

High-throughput TEM has also been used to study the effect of experimental parameters on the crystallinity and structure of MOFs (Fig. 11).^255^ Moreover, cryo-TEM has been applied to observe the initial formation of amorphous phases prior to the synthesis of cMOFs, which can be used to inform parameters for the direct synthesis of aMOFs.^51^ However, the application of TEM to MOFs is still limited, primarily because of the susceptibility of MOFs to beam damage, as well as the time-intensive sample preparation.^252,256,257^ Application of the electron beam to a MOF sample often results in rapid crystallinity loss by structural collapse, inducing amorphisation in its own right.^252,256^ To circumvent this, low beam currents can be applied, but this limits the data quality. To improve the reduced data quality, energy filtering can be applied to TEM, reducing the effect of unwanted inelastic electron scattering and producing a more interpretable image.^258^

The use of scanning electron microscopy (SEM) to investigate the presence of defined crystal facets within MOF is common.^68^ Whilst there are examples within the literature of clear morphology changes upon crystallisation,^130,201,259^ lack of defined crystal facets within the product is insufficient to conclude that a topologically amorphous material has been prepared. In addition to being likely indistinguishable from a poorly crystalline equivalent, highly defective MOF samples have also been observed to have incredibly distorted crystal facets.^260,261^ Because of this, sole reliance on SEM to draw conclusions about the sample crystallinity would likely lead to incorrect conclusions being drawn.

In addition to diffraction and microscopy techniques, thermal analysis has also been used to confirm or speculate on the presence of an amorphous phase. Differential scanning calorimetry (DSC) shows the glass transition temperature of a glass material. Whilst glasses are a type of amorphous phase, not all amorphous materials are glasses. The presence of this thermal transition is a feature of only glassy states and can therefore not be used to distinguish non-glassy amorphous phases from nanocrystalline ones. The formation of melt-quenched MOF glasses can be monitored with DSC, but additional techniques are required to confirm the amorphous structure of the sample.^92,97,262^ TGA can be used to evaluate the decomposition temperature of a material. Whilst not a quantitative measure, the lack of crystallinity would likely result in a decrease in thermal stability of a MOF, as with other materials. A decrease in decomposition temperature is therefore a potential suggested metric for which to determine the presence of an amorphous material. This, however, is likely to be highly inaccurate because of the number of parameters which control thermal stability, with an additional inability to differentiate the level of disorder within the sample. Due to these challenges, crystallographic techniques are key to confirming a lack of order within the sample.

### 5.2. SRO analysis

Despite the insights that that X-ray, electron, and heating-based techniques provide on disordered structures, studying the short-range order often requires additional techniques. Utilising a range of techniques for structural analysis is especially important when investigating amorphous materials with no crystalline equivalent for structural comparison.^188^

Extended X-ray absorption fine structure (EXAFS) investigates the local chemical environments of MOFs, giving the number, type and distances of neighbouring atoms. This specifically provides information about the inorganic cluster and how it bonds to the linkers. EXAFS has the advantageous ability to probe local structure at the early stages of synthesis. Investigation of the local structure of an early-stage amorphous material during the crystalline synthesis of MIL-89 revealed consistency of the SRO to that of the resultant crystalline material.^128^ Ball-milling amorphisation of several common MOFs concluded that HKUST-1, ZIF-8 and MOF-74 retained their EXAFS profiles upon amorphisation, consistent with a retention in local structure.^263^ In contrast, EXAFS of ball-milled UiO-66 revealed distortion in the local structure compared to the crystalline material, inconsistent with other studies on this structure.^248,263^ For investigation of the thermally-facilitated crystalline-amorphous transition of Cu-MOF, Cu[Cu(pdt)_2_] (pdt = 2,3-pyrazinedithiolate), a change in EXAFS profiles, and therefore local coordination, was observed upon formation of the aMOF.^264^ Distortion of the N–Cu–N bonds, as well as an increase in Cu coordination number upon amorphisation, was observed as a result of new Cu–S bonds forming.^264^ EXAFS was additionally utilised to confirm that the local structure of ZIF-8 was retained upon amorphisation, occurring upon enzyme incorporation during the synthetic procedure.^125^

Alternatively, electron paramagnetic resonance (EPR) can be used to give information on the electronic state and the coordination environment of a paramagnetic species, which are often present within the metal of the SBU. For example, EPR spectroscopy identified changes in the metal-coordination upon pressure-induced amorphisation of ZIF-8.^265^ This change was consistent with PDF studies of the same material, when exposed to equivalent conditions.^266^

Additionally, X-ray photoelectron spectroscopy (XPS) provides insights into the binding environment of an element. Investigation of MOF-74-Cu revealed equivalent coordination properties of both the crystalline and amorphous phases.^142^ An XPS study of both crystalline and amorphous UiO-66-NO_2_ revealed that a high proportion of linker defects were produced through a reduction in the number of Zr-acetate bonds.^207^ The range of techniques utilised to study both the short-range order and the general level of order within the material is promising, but determining which combination of these are required to make concrete conclusions about an aMOF material is still to be determined.

## 6. Future perspectives

### 6.1. Synthetic considerations

As expected from the variation present in crystalline synthetic methodologies, there are no general modifications to the synthetic method which will reliably result in the formation of an amorphous MOF. This review provides an indication to the starting points for the preparation of a_s_MOFs. Lowering the reaction temperature, decreasing the volume of solvent, as well as potentially introducing water to the reaction mixture would all be valid places to start, although the effectiveness of these will likely vary depending on the metal salt and organic linker being utilised. The variation of synthetic methods for the preparation of different aMOFs in the literature highlights the lack of understanding of how the mechanism of formation is affected by reaction parameters. This is complicated by insufficient investigation into the generalised mechanism of MOF formation and how this varies with different precursors and synthetic conditions. A systematic study into the effect of different parameters on the crystallinity of a small number of archetypal MOFs would be beneficial for MOFs containing similar/the same linkers.

Investigation into the formation of zeolites, microporous aluminosilicates, could provide further valuable insights into the mechanism of formation of MOF materials. Although the composition of MOFs differs greatly from zeolites, several crystalline MOFs, such as ZIFs, form similar network structures. The potential for synthetic methods applicable to the preparation of other amorphous materials to be applied to MOFs has been demonstrated through the modification of the Stöber method, commonly applied to disordered SiO_2_ colloids, highlighting the advantages of looking to equivalent systems for inspiration. Zeolite synthesis is observed to progress through a variety of mechanisms, much like MOF formation, with rearrangement from an amorphous or disordered phase to a crystalline one most commonly noted.^267^ Modelling showed it was energetically favourable for nucleation to occur to a disordered material, likely something which persists with MOF synthesis.^268^ It was then observed that subsequent exposure to both ambient or elevated temperature conditions resulted in a disorder-order transition occurring.^269^ As with MOF formation, investigation into the structure present at the early stages of zeolite synthesis has differed depending on the material being investigated. This highlights the persistent challenge with modification of known synthetic methodologies to induce extreme sample disorder. Depending on the material, either amorphous precursors, with equivalent SRO to the crystalline material, or small crystallites forming directly from starting materials.^267,270^

Given the similarities in the potential mechanisms of formation of these materials to MOFs, inspiration could be drawn from the research into the effect of different synthetic parameters on zeolite crystallisation, including temperature, solvent and concentration.^271^ Additionally, information on how the nature of the material affects the nucleation pathway could be conferred onto control of MOF formation mechanisms.

Whilst this review does not provide explicit instructions for amorphous synthesis, combining the information provided with the knowledge about nucleation mechanisms provides an insight into how to control the kinetics of this formation. The formation of crystalline zeolite structures has additionally been observed to progress through an amorphous hydrogel intermediate, before subsequent crystallisation occurred.^272,273^ Whilst again underrepresented in the research, synthetic parameters of the gel-based synthesis of cMOFs have been previously explored, providing some additional indication of starting points for the preparation of aMOFs.

What is most promising with aMOF synthesis is, however, the seeming consistency of synthetic methodologies when varying the functionality of the linker. This would suggest that development of a reliable synthesis could be tracked across a series of aMOFs, likely with consistent metal salts. The literature understanding of defect engineering could also be applied to these disordered materials to control the level of unsaturation in the resultant aMOF. Controlling the level of both disorder and defects in a MOF structure presents additional ways to tune the functionality of these materials past that of varying the composition of the MOF.

### 6.2. Modelling

To date, little complete structural characterisation of aMOFs has been conducted, despite increases in frequency to which they have been reported. Because of this, development of structural models has been challenging, as no comparison could be made to either form the basis of additional models or validate the accuracy of existing ones. Some structural models utilise cMOF structural data to determine the resultant deformations that can occur, for example a model of aZIF-4 was developed, using known structural similarities to aSiO_2_.^55^ Modification of a continuous random network (CRN) model, ensuring no defects in the structure, produced PDF spectra equivalent to that of the experimental data.^274^ However, although electronic properties were also simulated, these were not compared to experimentally obtained values, leading to questions about the accuracy of these defect-free CRN models.^274^ Since defects are theorised to have a noteworthy effect on the properties of these materials, the ability to correctly model them within the structure would be significant to improving accuracy. Molecular dynamics (MD) simulations have also been utilised to predict the porosity of amorphous ZIF-8 upon guest exclusion from the pores, with the simulated radial distribution function matching well with the experimental data.^125^

Another example included altering a structure initially developed for amorphous polymers to be suitable for modelling Fe-BTC.^221,275^ From this model, porosity of different potential structures could be predicted and compared to experimental data for structure determination.^276^ This highlights the potential for predictive models to be developed to analyse the properties of new amorphous MOFs, which would be beneficial for the application-specific design of these materials.

Finally, the utilisation of limited amorphous structural models to train machine learned potentials (MLP) for the fast generation of new disordered models has been demonstrated.^277^ Providing an alternative to *ab initio* and density functional theory calculations, MLP allowed for not only glassy and amorphous ZIF-4 models to be generated, but also for further investigation of the processes involved with formation of the melt-quenched glass.^277^ Comparisons were made to experimental mechanical and structural data, demonstrating the accuracy of the developed models. This demonstrates the likely progression in the modelling of these disordered materials.^277^

Literature has reported the successful compilation of a database of porous amorphous materials, detailing not only the ability to model disordered materials, but also compute a range of physical properties, beneficial for database mining for specific applications.^278^ If achievable for aMOFs, this would present a significant opportunity to prepare a_s_MOFs for specific applications with a targeted approach.

### 6.3. Applied considerations

The previously discussed potential for either greener or less energy-intensive synthesis of a_s_MOFs, compared to the parent cMOF, could allow for wider industrial application through ease of scalability. This additionally highlights the benefits of direct synthesis, compared to that of post-synthetic amorphisation to form aMOFs. Limitations present with the stability of cMOFs, both thermodynamically as well as mechanically, are not present in a_s_MOF systems, increasing the range of potential MOFs which could be investigated for industrial applications. Direct synthesis allows easy and quick preparation of aMOF materials, with the potential to control the functionality, structure, and disorder of the product for specific desired properties. Additionally, direct synthesis would potentially allow for tuning of the missing linker or cluster ‘defects’ of these materials, similar to that currently carried out on cMOFs, through variation of synthetic parameters.^44,59,215,217,218,220^ In general, the intrinsically defective MOF systems present improved properties for several functionalities, resulting in a wide scope for potential applications, highlighting the potential breadth of applications available for new aMOF systems.

## 7. Conclusions

The fabrication of amorphous MOFs and the understanding of the synthetic parameters for their successful preparation will open exciting avenues to implement these materials in the industry. Subsequent full characterisation and prediction of the structure and properties of aMOF materials will allow for increased investigation into disordered phases going forward. Their ease of tunability and low energy-cost synthesis would also allow further applications to be investigated efficiently. Understanding and controlling how both disorder and defects affect the properties of these materials would introduce additional parameters to be tuned for desired applications. Consistency in the characterisation of these materials, allowing true identification of sample disorder, would allow trends in synthetic conditions to be identified, progressing the development of transferable synthetic methods. Utilising the characterisation techniques outlined here, a full understanding of the progression of structure from amorphous to crystalline could be developed. This could then allow for more accurate systematic design of disordered materials. Given the enormous promise for the synthesis and characterisation of thousands of further directly synthesised topologically amorphous MOF samples, we would predict that, with appropriate care with characterisation, this field will continue to grow and produce materials that can challenge the dominancy of the crystalline domain.

## Abbreviations

aMOF: Amorphous metal–organic framework
BDC: Benzene dicarboxylic acid
BET: Brunauer–Emmett–Teller
bIm: Benzimidazole
BTB: 1,3,5-Benzenetrisbenzoate
BTC: 1,3,5-Benzenetricarboxylic acid
CAU: Christian-Albrechts-Universität
cMOF: Crystalline metal–organic framework
CRN: Continuous random network
DCIM: 4,5-Dichloroimidazolate
DES: Deep eutectic solvent
DMA: Dimethylacetamide
DMF: Dimethylformamide
DOBDC: 2,5-Dioxido-1,4-benzenedicarboxylic acid
DOT: Dioxidoterephthalate
DSC: Differential scanning calorimetry
EPR: Electron paramagnetic resonance
EXAFS: Extended X-ray absorption fine structure
HBITP: 2,6-Bis-(4-imidazole-1-yl-phenyl)-4-[4-(2*H*-tetrazol-5-yl)phenyl]-pyridine
H_2_BPDC: Biphenyl-4,4-dicarboxylic acid
Hcam: (+)-Camphoric acid
H_4_DHTA: 2,5-Dihydroxy-1,4-benzenedicarboxylic acid
H_2_DHTA: 2,5-Dihydroxyterephthalic acid
H_2_fipbb: 4,4′-(Hexafluoroisopropylidene)bis(benzoic acid)
H_2_IBTP: 4-(4-(1*H*-Imidazole-1-yl)-phenyl)-2,6-bis(4-(2*H*-tetrazol-5-yl)phenyl)pyridine
H_2_IPA: Isophthalic acid
HKUST: Hong Kong University of Science and Technology
H_2_PMDA: *N*,*N*′-Pyromelliticdiimido-di-l-alanine
H_2_SDA: 4,4′-Stilbenedicarboxylic acid
ICA: Imidazole-2-carboxyaldehyde
Im: Imidazole
LAG: Liquid assisted grinding
LRO: Long-range order
MD: Molecular dynamics
MeOH: Methanol
mIm: Methyl imidazole
MIL: Materials of institute lavoisier
MLP: Machine learned potentials
MQG: Melt-quenched glass
MOF: Metal–organic framework
PDF: Pair distribution function
PDT: Pyrazinedithiolate
PPD: *p*-Phenylenediamine
PNC: Pre-nucleation cluster
PXRD: Powder X-ray diffraction
SBU: Secondary building unit
SDA: Structure directing agent
SEM: Scanning electron microscopy
SRO: Short-range order
STEM: Scanning transmission electron microscopy
*T*_d_
: Decomposition temperature
TEA: Triethylamine
TEM: Transmission electron microscopy
*T*_g_
: Glass transition temperature
TGA: Thermogravimetric analysis
*T*_m_
: Melting temperature
UiO: Universitetet i Oslo
XPS: X-ray photoelectron spectroscopy
ZIF: Zeolitic imidazolate framework

## Data availability

No primary research results, software or code have been included and no new data were generated or analysed as part of this review.

## Author contributions

The draft manuscript was prepared by E. V. S. All authors contributed to the final manuscript.

## Conflicts of interest

There are no conflicts to declare.
